# Transcriptomic analysis reveals that neural stem cell-derived exosomes regulate the HMGB1/TLR2 signaling axis to promote astrocytic differentiation and mitochondrial biogenesis in the repair of radiation-induced blood-brain barrier damage

**DOI:** 10.1038/s41420-026-03250-4

**Published:** 2026-07-25

**Authors:** Fanrui Zeng, Yalei Zhang, Yun Zhou, Zichen Ma, Chenghao Li, Guohua Yao, Rong Li

**Affiliations:** 1 Department of Radiation Oncology, The First Affiliated Hospital of Guangzhou Medical University, Guangzhou, China; 2 Department of Thoracic Oncology, The First Affiliated Hospital of Guangzhou Medical University, Guangzhou, China; 3 Guangzhou Medical University, Guangzhou, China; 4 Jiangxi University of Traditional Chinese Medicine, Nanchang, China; 5 Department of Medical Oncology, The First Affiliated Hospital of Guangzhou Medical University, Guangzhou, China

**Keywords:** Blood-brain barrier, Molecular biology

## Abstract

Radiation-induced brain injury (RBI) is frequently associated with blood–brain barrier (BBB) disruption, which contributes to poor prognosis. Neural stem cell-derived exosomes (NSC-Exo) have recently attracted attention as mediators of intercellular communication with potential roles in tissue repair. However, how NSC-Exo promote BBB recovery after RBI remains unclear. This study explored whether NSC-Exo restore BBB function by regulating the HMGB1/TLR signaling pathway, thereby promoting endogenous NSC differentiation toward astrocytes and enhancing mitochondrial biogenesis. A rat RBI model was established and treated with NSC-Exo. Molecular marker analysis, transcriptomic profiling, and BBB functional assessment were performed to clarify the underlying mechanisms. The results showed that NSC-Exo enhanced NSC stemness and astrocytic differentiation, improved mitochondrial function, and reduced ROS accumulation. NSC-Exo also suppressed HMGB1/TLR2 pathway activation and promoted BBB repair. Functional experiments further indicated that HMGB1 overexpression weakened the protective effects of NSC-Exo, whereas TLR2 knockdown reversed this effect. In conclusion, NSC-Exo facilitate endogenous NSC remodeling and BBB restoration after RBI, at least partly through regulation of the HMGB1/TLR2 axis, providing a potential strategy for RBI treatment.

**Figure:**
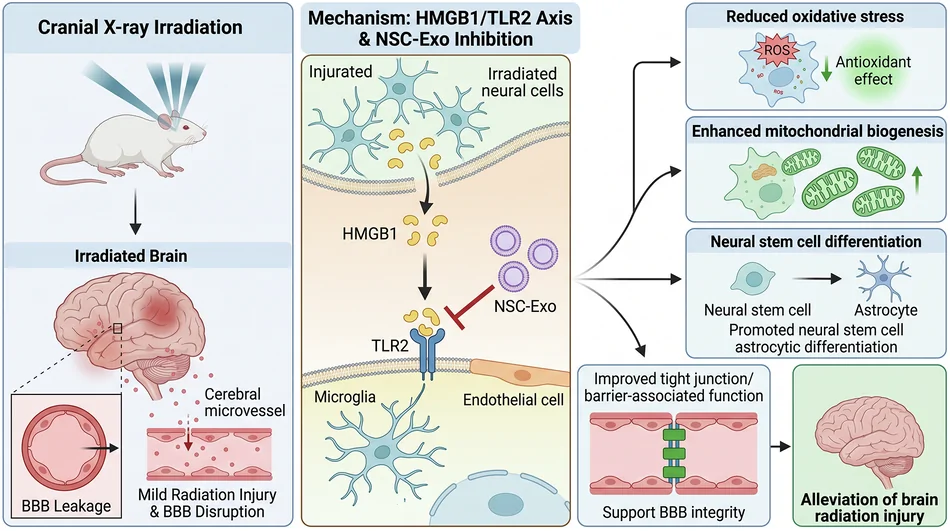
Schematic illustration of the molecular mechanism by which NSC-Exo ameliorates RBI through inhibition of the HMGB1/TLR2 signaling pathway.

Schematic illustration of the molecular mechanism by which NSC-Exo ameliorates RBI through inhibition of the HMGB1/TLR2 signaling pathway.

## Introduction

Radiation-induced brain injury (RBI) is a frequent adverse effect of tumor radiotherapy and often leads to neurological dysfunction, cognitive decline, and impaired quality of life [1–3]. Ionizing radiation can induce multifaceted structural and functional damage in brain tissue, including cellular apoptosis, neuroinflammatory responses, increased production of free radicals, and disruption of microenvironmental homeostasis [4]. Among these, damage to the blood-brain barrier (BBB) is considered a key event in the progression of RBI [5]. Disruption of the BBB exposes brain tissue to harmful molecules and immune cell infiltration, exacerbating inflammation and neuronal injury [5, 6]. Currently, there is a lack of effective pharmacological agents or approaches specifically targeting BBB repair, and patient outcomes remain suboptimal [7, 8]. Therefore, clarifying the mechanisms of BBB injury and repair in RBI is essential for developing new therapeutic approaches.

Clinically, radiation-induced brain injury evolves across acute, early-delayed, and late phases, and late effects are often linked to persistent neuroinflammation, vascular rarefaction, demyelination, and impaired neurogenesis [9, 10]. These time-dependent changes make BBB dysfunction not only an early manifestation of tissue injury but also a driver of chronic microenvironmental instability after cranial radiotherapy [11, 12].

In recent years, neural stem cells (NSCs), owing to their self-renewal and multipotent differentiation capabilities, have demonstrated significant potential in the repair of nervous system injuries [13, 14]. NSCs contribute to tissue regeneration and microenvironmental regulation through the secretion of various bioactive molecules; however, direct cell transplantation is associated with challenges such as immune rejection, low engraftment efficiency, and safety concerns [15]. Exosomes are nanoscale vesicles secreted by cells that carry proteins, lipids, and nucleic acids, and mediate intercellular communication [16]. NSC-derived exosomes (NSC-Exo), with their high safety profile and low immunogenicity, are increasingly recognized as a promising “cell-free” strategy for the intervention of brain injury [17, 18]. While extensive literature [17] has documented that NSC-Exo promote neuronal survival, suppress inflammation, and modulate neuroregeneration in other contexts, their specific molecular mechanisms in BBB repair—particularly in the context of RBI—remain insufficiently understood.

Cell-free vesicle therapies have been explored in several neurological injury models because extracellular vesicles can deliver regulatory RNAs, proteins, and lipids while avoiding some risks associated with cell transplantation. Studies of NSC-derived extracellular vesicles indicate that their neuroprotective effects may involve anti-inflammatory signaling, support of neuronal survival, and modulation of mitochondrial metabolism, although these effects vary by disease context and vesicle cargo composition [19, 20].

In RBI and other neurological diseases, inflammatory responses are important contributors to BBB disruption and neuronal damage [21, 22]. High mobility group box 1 (HMGB1), a key inflammatory mediator, is released in response to cellular stress and injury. By activating signaling pathways such as Toll-like receptor 2 (TLR2) and the receptor for advanced glycation end products (RAGE), HMGB1 regulates the release of inflammatory cytokines and the induction of cell apoptosis. The HMGB1/TLR2 signaling axis not only plays a pivotal role in neuroinflammation but also influences stem cell fate and the maintenance of microenvironmental homeostasis [23]. In addition, HMGB1 can upregulate the expression of matrix metalloproteinase-9 (MMP-9), promote degradation of the basement membrane of the BBB, thereby increasing BBB permeability and leading to brain edema and inflammatory cell infiltration [24]. Previous studies have demonstrated that, in models of RBI, activation of HMGB1 and TLR2 is significantly increased. This heightened activation results in increased BBB permeability and an aggravated inflammatory response, which further exacerbates brain injury [25, 26]. As a result, regulation of the HMGB1/TLR2 signaling axis has become an important direction in brain injury repair. Exosomes, as emerging mediators of intercellular communication, present a new avenue of investigation regarding whether modulation of HMGB1/TLR2 signaling by exosomes can promote NSC differentiation and facilitate BBB repair.

HMGB1 biology is multifaceted: extracellular HMGB1 can engage TLR2, TLR4, and RAGE, and this receptor diversity links sterile inflammation to endothelial injury, microglial activation, and BBB permeability in brain injury models [27, 28]. Therefore, focusing on HMGB1/TLR2 provides a tractable axis for mechanistic testing while related HMGB1-dependent pathways may also contribute to RBI pathology.

Mitochondria are essential for NSCs to maintain their function and achieve differentiation, as their biogenesis and functional status directly affect the survival, proliferation, and differentiation potential of NSCs [29–31]. Radiation exposure disrupts mitochondrial homeostasis, leading to reduced membrane potential, impaired ATP production, and excessive reactive oxygen species (ROS) generation. These changes disturb cellular energy metabolism and increase oxidative stress, ultimately hindering neural repair [32]. Recent research has shown that promoting mitochondrial biogenesis and maintaining mitochondrial stability can improve the physiological state of neural cells and enhance their resistance to injury [33, 34]. In addition, exosomes have been shown to regulate mitochondrial activity in various pathological conditions by delivering specific microRNAs and proteins. Based on this, the present study aims to investigate the molecular mechanisms by which NSC-Exo regulate mitochondrial biogenesis and the differentiation of endogenous NSCs in the context of RBI. Further research in this area is crucial for advancing the theoretical framework for the repair of RBI.

These observations place NSC-Exo within a broader literature in which extracellular vesicles influence mitochondrial quality control and intercellular metabolic support. The specific contribution of NSC-Exo to mitochondrial remodeling and astrocytic differentiation after RBI, however, remains incompletely defined.

Building on this background, this study utilizes a rat model of RBI to investigate the involvement and molecular mechanisms of exogenous NSC-Exo in BBB repair. The study focuses on whether NSC-Exo promotes the differentiation of endogenous NSCs into astrocytes and enhances mitochondrial biogenesis by regulating the HMGB1/TLR2 signaling pathway. Using Western blot (WB), immunofluorescence, flow cytometry, and transcriptomic analyses, the results demonstrate that NSC-Exo treatment significantly upregulates stemness and differentiation markers in NSCs, improves mitochondrial function, reduces the production of ROS, and restores BBB function. Moreover, NSC-Exo markedly inhibits the activation of the HMGB1/TLR2 signaling axis. Further molecular intervention experiments confirm the key regulatory role of this pathway. Collectively, this study provides new theoretical insights and molecular targets for BBB repair and the treatment of RBI.

## Results

### NSC-Exo improve cognitive function and promote BBB repair in rats with RBI

To verify the extraction and characterization of NSC-Exos, a comprehensive analysis was conducted. The exosome isolation procedure is shown in Fig. S1A. TEM revealed that the isolated exosomes exhibited the characteristic cup-shaped, double-membrane structure (Fig. S1B). NTA indicated that the particle size was primarily distributed between 100 and 150 nm, with a uniform size distribution (Fig. S1C). WB further confirmed the expression of exosomal marker proteins Alix, Hsp90, CD63, and Tsg101, while the negative marker Calnexin was not detected (Fig. S1D), indicating a high degree of exosome purity.

To evaluate the cellular and in vivo uptake of these exosomes, Exo-Green-labeled exosomes were co-cultured with HNCs and NSCs for 24 h (Fig. S2A). Confocal microscopy showed green fluorescence signals in both cell types, indicating successful internalization of exosomes by the cells (Fig. S2B). In addition, MTT assay results showed that exosome treatment did not significantly affect cell viability (Fig. S2C).

Subsequently, in vivo fluorescent imaging (IVIS) was used to dynamically track the distribution of Exo-Green-labeled exosomes after intravenous injection via the tail vein in rats. The results showed that fluorescent signals in the brain region appeared as early as 30 min post-injection and continued to increase over 360 min (Fig. S2D), suggesting that the exosomes have the capacity to cross the BBB.

**Fig. 1:**
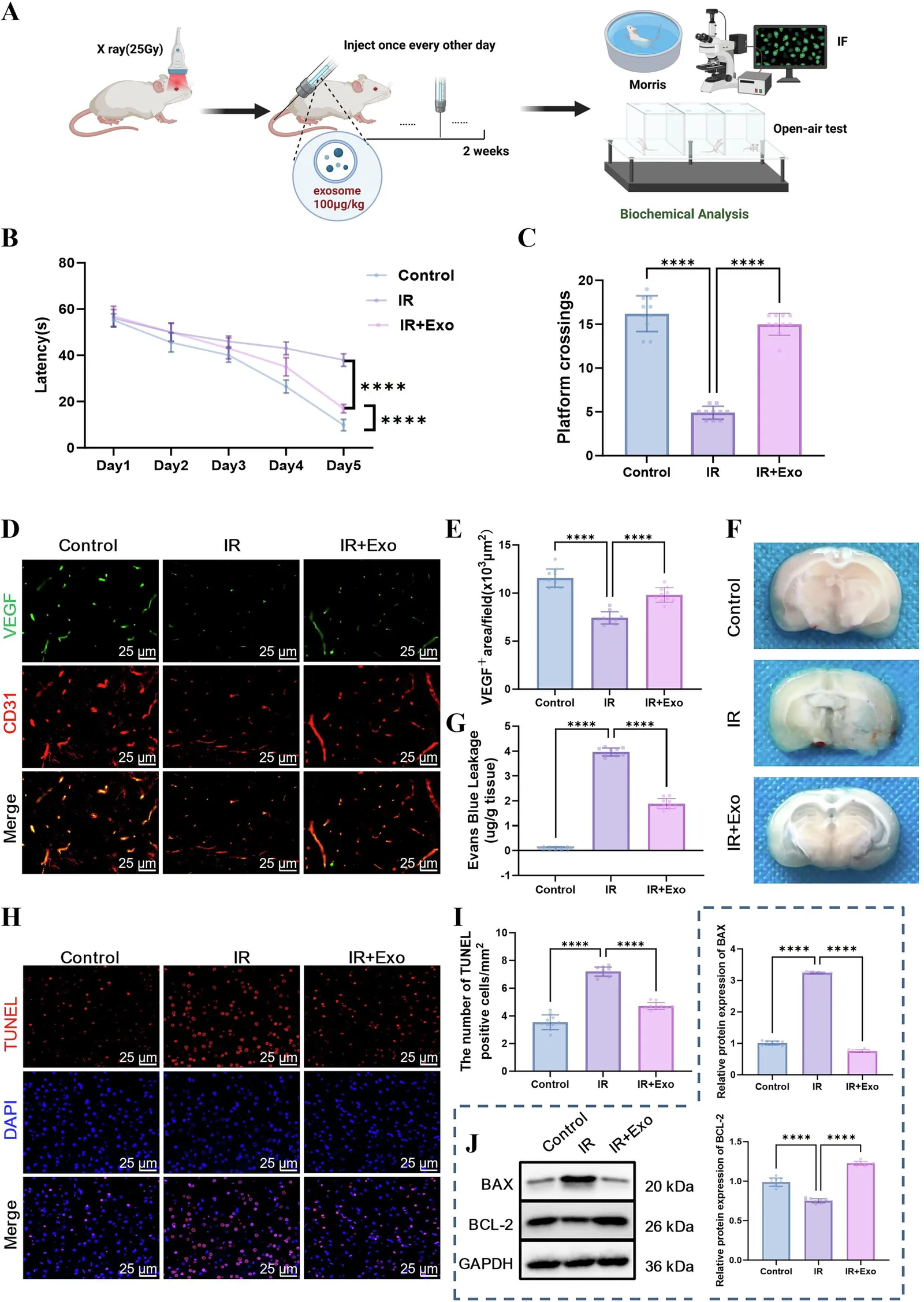
NSC-Exo improve cognitive function and promote BBB repair in rats with RBI. **A** Schematic diagram of the experimental protocol showing exosome-mediated amelioration of RBI in rats; **B** Morris water maze test assessing cognitive function by measuring escape latency; **C** Morris water maze test evaluating cognitive function by the number of platform crossings; **D**, **E** Immunofluorescence analysis of neovascular density in the irradiated brain region (bar = 25 μm); **F**, **G** Evans blue staining for BBB permeability in rat brain tissue (bar = 5 mm); **H**, **I** TUNEL staining detecting neuronal apoptosis in irradiated brain tissue (bar = 25 μm); **J** Western blot analysis of BAX and BCL-2 protein expression in brain tissue. Each group contained 10 rats; data are presented as mean ± standard deviation. **p < 0.01, ***p < 0.001, ****p < 0.0001.

To further evaluate the effects of NSC-Exos in a rat model of RBI, exosomes were administered via tail vein injection following 25 Gy X-ray irradiation (Fig. 1A). In the Morris water maze test, the IR group showed significantly prolonged escape latency compared with the control group, whereas exosome treatment markedly shortened this latency (Fig. 1B). In the probe trial, platform crossings were significantly reduced in the IR group but were restored after exosome administration (Fig. 1C).

Immunofluorescence analysis further demonstrated that, relative to controls, the density of newly formed cerebral microvessels was decreased in the IR group. In contrast, exosome-treated rats exhibited enhanced CD31- and VEGF-positive signals in the injured area (Fig. 1D, E). Evans blue staining demonstrated increased dye permeability in the IR group, whereas the amount of dye permeation was significantly decreased following exosome treatment (Fig. 1F, G), indicating a potential role in maintaining BBB integrity.

TUNEL staining revealed a significant increase in apoptotic cells in the brain tissue of the IR group, whereas exosome treatment markedly reduced the number of TUNEL-positive cells (Fig. 1H, I). WB analysis further showed that BCL-2 expression was decreased in the IR group but was restored following exosome administration. In contrast, BAX expression was elevated after irradiation and subsequently reduced by exosome treatment (Fig. 1J).

These observations reveal that NSC-Exos can be taken up by neuro-associated cells and enter brain tissue in vivo, suggesting their potential regulatory roles in cognitive function maintenance, BBB protection, angiogenesis, and anti-apoptotic processes.

### The key role of the HMGB1/TLR2 axis in mitochondrial homeostasis and inflammatory response during exosome-mediated alleviation of RBI

**Fig. 2:**
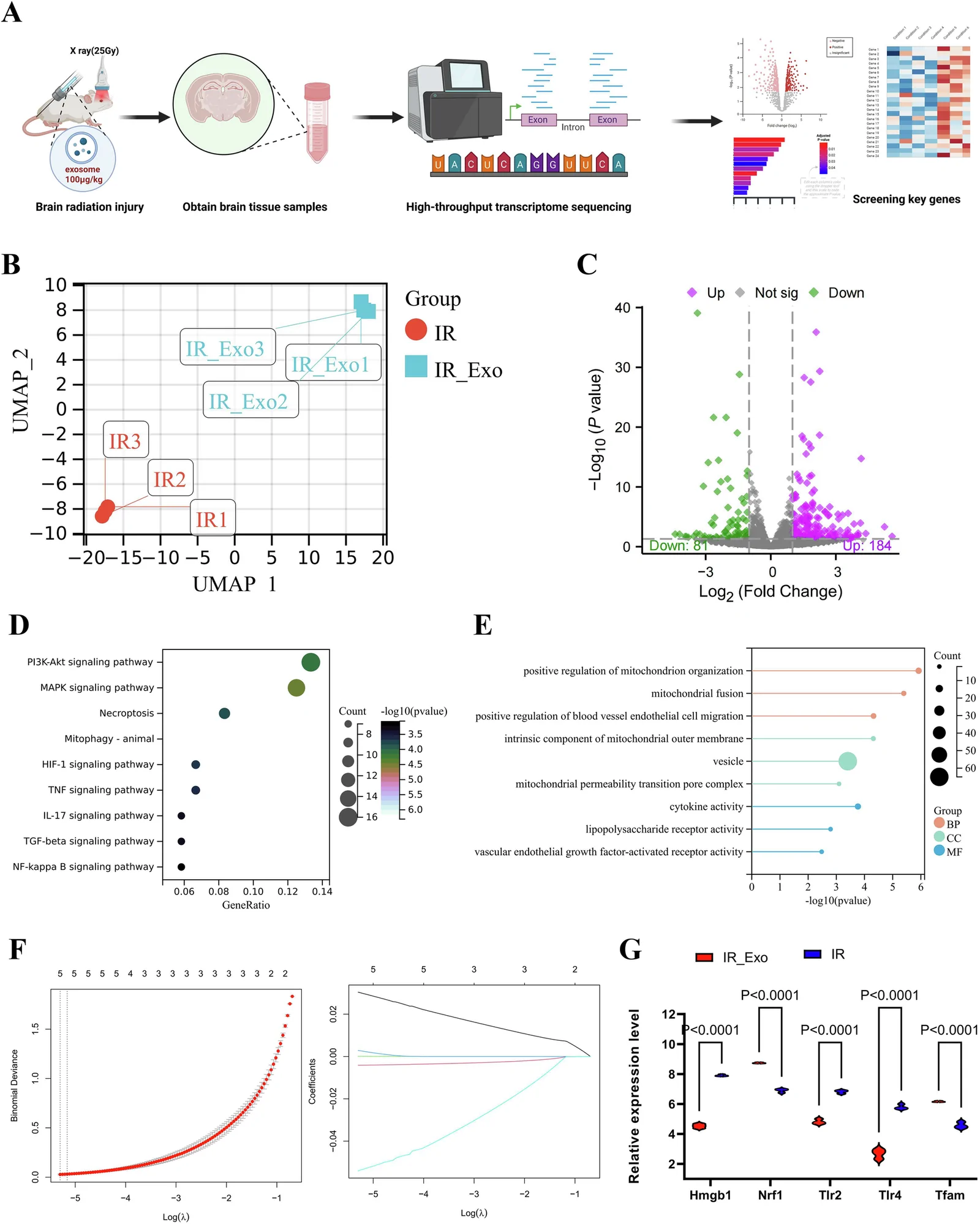
Transcriptomic analysis of exosome intervention in alleviating RBI. **A** Schematic diagram of the experimental workflow: collection of brain tissue samples, high-throughput transcriptomic sequencing, and identification and enrichment analysis of DEGs; **B** UMAP dimensionality reduction plot showing clear separation at the transcriptomic level between the IR (red) and IR_Exo (blue) groups; **C** Volcano plot of DEGs, identifying 184 upregulated and 81 downregulated genes (selection criteria: |log₂FC | > 1, *p* < 0.05); **D** KEGG pathway enrichment bubble plot, displaying significantly enriched pathways and associated gene counts and significance; **E** GO BP enrichment analysis, showing biological functional categories related to DEGs, with color and dot size representing group and gene count, respectively; **F** LASSO regression analysis, with the left panel showing the cross-validation curve and the right panel displaying the distribution of gene coefficients as the regularization parameter changes; **G** Expression level comparison of feature DEGs identified by LASSO analysis. IR: *n* = 3; IR_Exo: *n* = 3.

To investigate the protective mechanisms of exosomes in RBI, high-throughput transcriptomic sequencing was performed on brain tissue samples from the IR group and the exosome intervention group (IR_Exo) after RNA extraction (Fig. 2A). UMAP analysis demonstrated clear separation between the two groups, indicating distinct global gene expression profiles (Fig. 2B).

Differential expression analysis identified 265 DEGs, including 184 upregulated and 81 downregulated genes ( | log_2_FC | > 1, *P* < 0.05), as shown in the volcano plot (Fig. 2C). KEGG pathway enrichment revealed that these DEGs were mainly involved in pathways related to cell survival, apoptosis, and metabolism, including PI3K-Akt, MAPK, necroptosis, mitophagy, and TNF signaling pathways (Fig. 2D), indicating that exosomes may exert protective effects by regulating cellular stress and apoptotic mechanisms.

Additionally, GO analysis for BP and MF demonstrated that the DEGs were significantly enriched in functional modules such as “regulation of mitochondrial organization,” “mitochondrial fusion,” “mitochondrial membrane transition pore complex,” “regulation of endothelial cell migration,” and “cytokine activity” (Fig. 2E). These findings indicate that exosome intervention may play a potential role in maintaining mitochondrial function and modulating the neuroimmune microenvironment.

To identify potential key regulatory factors, a LASSO regression model was applied to the DEGs for feature selection, constructing a predictive model and identifying candidate genes strongly associated with the intervention response (Fig. 2F). Ultimately, key genes including HMGB1, MT1, TLR2, TNF, and TFAM were identified, all of which were significantly downregulated in the IR_Exo group. Among them, HMGB1 showed the highest statistical significance, suggesting that it may serve as the core regulatory factor in the exosome-mediated protective effects against RBI (Fig. 2G).

The above functional enrichment analyses revealed a close association with mitochondrial processes. Heatmap analysis showed that, in the IR_Exo group, several key mitochondrial protective factors (such as MFN1, MFN2, TFAM, and NRF1) were upregulated, while markers indicative of mitochondrial damage and mitophagy (such as BNIP3, PPIF, and PINK1) were significantly downregulated (Fig. S3A). These findings suggest that exosome intervention helps maintain mitochondrial structural stability and functional integrity.

Further construction of gene co-expression networks revealed that HMGB1 and TLR2 were highly correlated with several mitochondrial functional factors (such as TFAM, MFN1, NRF1, and ATG7), exhibiting a complex pattern of interactions. The HMGB1/TLR2 axis showed a positive correlation with mitochondrial damage factors (such as PINK1 and BNIP3) and a negative correlation with mitochondrial protective factors (such as TFAM and NRF1) (Fig. S3B), suggesting that this axis may promote mitochondrial dysfunction in the context of radiation injury.

PPI networks constructed based on the STRING database further confirmed that HMGB1 and TLR2 can regulate key mitochondrial homeostasis regulators, including PINK1, MFN1, MFN2, and ATG7, thereby participating in energy metabolism and cell fate determination after radiation injury (Fig. S3C). This finding highlights a potential crosstalk mechanism between immune responses and mitochondrial function.

Based on these findings, we propose a potential mechanistic model (Fig. S3D): Following X-ray-induced brain radiation injury, upregulation of HMGB1/TLR2 signaling activates pro-inflammatory pathways and inhibits mitochondrial fusion. Exosome intervention effectively suppresses HMGB1 and TLR2 expression, thereby relieving their inhibitory effects on mitochondrial function, promoting mitochondrial remodeling and tissue repair, and ultimately alleviating RBI. To validate the reliability of the transcriptomic findings, we further verified the expression of other DEGs such as TNF and MT1 by qPCR. The results showed that their expression trends were consistent with the transcriptomic data (Fig. S3E).

These results demonstrate the pivotal role of the HMGB1/TLR2 axis in RBI and clarify that exosomes exert their protective effects by modulating inflammation and mitochondrial homeostasis through regulation of this axis. Exosomes downregulate HMGB1 and its downstream inflammatory pathways, reverse radiation-induced impairment of mitochondrial fusion, and restore homeostasis in the neural microenvironment.

### Mechanistic role of the HMGB1/TLR2 axis in exosome-mediated attenuation of radiation-induced neuronal apoptosis

**Fig. 3:**
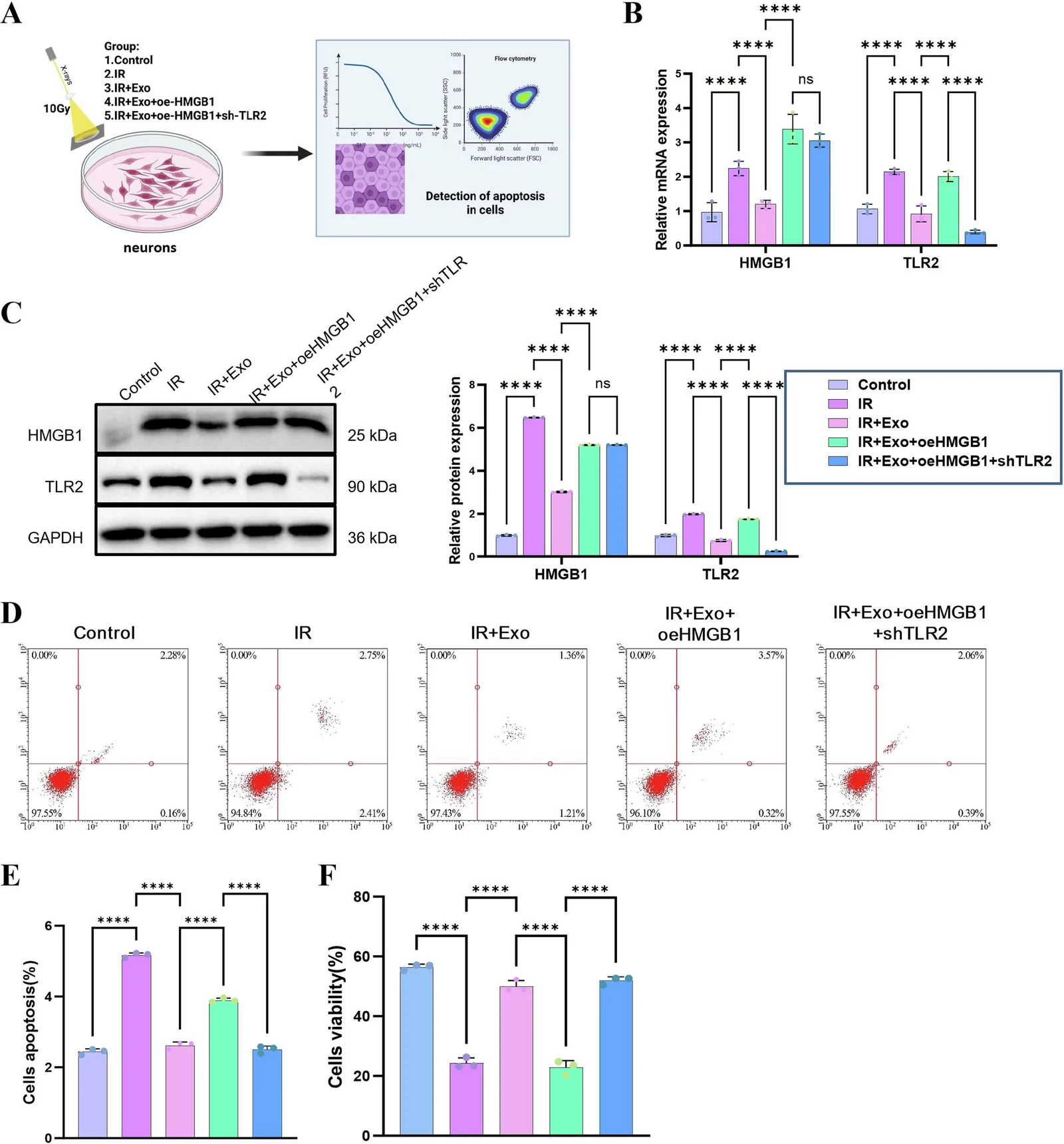
Exosomes attenuate radiation-induced neuronal apoptosis by modulating the HMGB1/TLR2 axis. **A** Schematic diagram of experimental groups and protocol; qRT-PCR (**B**) and Western blot (**C**) analyses of HMGB1 and TLR2 expression levels in different groups; **D**, **E** Annexin V/PI double staining and flow cytometry to detect cell apoptosis; **F** MTT assay for evaluating the cell viability of radiation-injured neurons. All cell experiments were repeated three times. Data are presented as mean ± standard deviation. *p < 0.05, **p < 0.01, ***p < 0.001, ****p < 0.0001.

To clarify the involvement of the HMGB1/TLR2 pathway in exosome-mediated repair of RBI, an in vitro neuronal radiation injury model was established. After exposure to 10 Gy of radiation, neurons were subjected to various interventions, resulting in five experimental groups: normal control (Control), IR, exosome intervention (IR + Exo), exosome plus HMGB1 overexpression (IR + Exo + oe-HMGB1), and exosome plus HMGB1 overexpression combined with TLR2 knockdown (IR + Exo + oe-HMGB1 + sh-TLR2) (Fig. 3A).

RT-qPCR and WB analyses showed that HMGB1 and TLR2 were significantly upregulated in the IR group, indicating activation of this inflammatory pathway after irradiation. Exosome treatment markedly suppressed the expression of both proteins. However, HMGB1 overexpression reversed this effect, restoring elevated levels of HMGB1 and TLR2. Subsequent TLR2 knockdown significantly reduced TLR2 expression without affecting HMGB1 levels, suggesting that HMGB1 functions upstream to regulate TLR2 (Fig. 3B, C).

Annexin V-FITC/PI staining (Fig. 3D, E) and MTT assays (Fig. 3F) were performed to evaluate apoptosis and cell viability, respectively. Flow cytometry showed a marked increase in neuronal apoptosis in the IR group, which was significantly alleviated by exosome treatment. HMGB1 overexpression enhanced apoptosis, whereas TLR2 knockdown attenuated this effect. Consistently, MTT results confirmed that the pro-survival effect of exosomes is mediated through the HMGB1/TLR2 axis.

TUNEL staining further assessed DNA fragmentation. In line with the flow cytometry and viability results, exosome treatment significantly reduced the number of TUNEL-positive cells compared with the IR group. HMGB1 overexpression markedly increased TUNEL staining, whereas TLR2 knockdown led to a significant reduction (Fig. S4A, B), confirming the critical role of TLR2 in HMGB1-mediated radiation-induced apoptosis.

Together, these findings demonstrate that exosome intervention effectively alleviates radiation-induced neuronal apoptosis, and this effect likely depends on the inhibition of the HMGB1/TLR2 signaling pathway.

### Exosomes improve radiation-induced mitochondrial dysfunction in neurons by inhibiting the HMGB1/TLR2 signaling axis

**Fig. 4:**
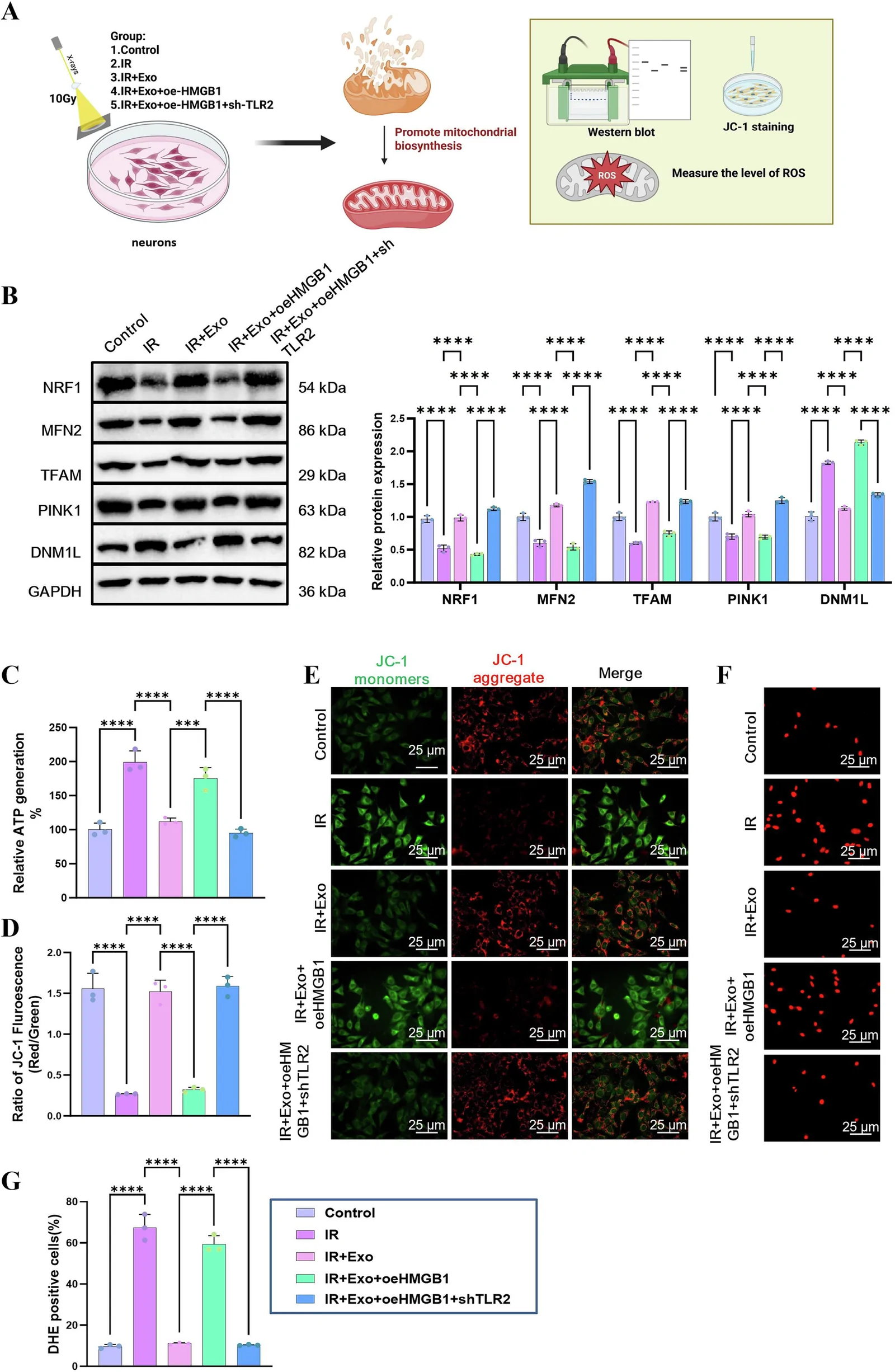
Exosomes regulate mitochondrial function and improve energy metabolic homeostasis in radiation-injured neurons via inhibition of the HMGB1/TLR2 axis. **A** Schematic diagram of the experimental protocol; **B** Western blot analysis of mitochondrial-related protein expression in radiation-injured neurons; **C** ATP colorimetric assay quantifying ATP production as a percentage in radiation-injured neurons; **D**, **E** JC-1 staining to evaluate mitochondrial membrane potential in radiation-injured neurons (bar = 25 μm); **F**, **G** DHE staining to detect ROS levels in radiation-injured neurons (bar = 25 μm). All cell experiments were repeated three times. Data are presented as mean ± standard deviation. *p < 0.05, **p < 0.01, ***p < 0.001, ****p < 0.0001.

Transcriptomic analysis revealed that DEGs following exosome intervention are closely associated with mitochondrial function, and that the HMGB1/TLR2 pathway interacts significantly with several mitochondrial regulatory factors. To determine whether exosomes modulate mitochondrial function through this pathway, further in vitro mechanistic studies were conducted in neuronal cells (Fig. 4A).

WB analysis showed that, in the radiation injury group, the expression of proteins promoting mitochondrial fusion and biogenesis (NRF1, MFN2, TFAM, PINK1) was significantly decreased, while the expression of the mitochondrial fission and damage-associated protein DNM1L was upregulated. Exosome intervention (IR+Exo) reversed these trends, suggesting that exosomes enhance mitochondrial fusion and inhibit excessive fission. Overexpression of HMGB1 attenuated these protective effects, whereas TLR2 knockdown partially restored the regulatory effects of exosomes on mitochondria (Fig. 4B). These results indicate that exosomes improve radiation-induced mitochondrial dysfunction by inhibiting the HMGB1/TLR2 pathway.

At the level of mitochondrial energy metabolism, ATP assays showed a significant decrease in ATP levels in the IR group, whereas exosome treatment restored ATP production to near-normal levels. HMGB1 overexpression suppressed ATP generation again, while TLR2 knockdown partially rescued ATP levels (Fig. 4C), indicating that the HMGB1/TLR2 axis is involved in regulating cellular energy metabolism.

JC-1 staining was used to evaluate changes in mitochondrial membrane potential. The red/green fluorescence ratio was significantly reduced in the IR group, indicating impaired membrane potential. Exosome treatment markedly increased membrane potential. HMGB1 overexpression again led to decreased membrane potential, while the addition of TLR2 knockdown restored the red aggregate fluorescence (Fig. 4D, E), demonstrating that exosomes can stabilize membrane potential via the HMGB1/TLR2 axis.

DHE staining further evaluated mitochondrial ROS levels. The IR group exhibited elevated ROS-associated fluorescence, indicating increased oxidative stress, while exosome treatment significantly reduced ROS accumulation. Overexpression of HMGB1 abolished this protective effect, whereas TLR2 knockdown restored the capacity for ROS clearance (Fig. 4F, G), further confirming the central role of the HMGB1/TLR2 pathway in ROS regulation.

Collectively, exosomes downregulate the HMGB1/TLR2 signaling axis, modulate the expression of key mitochondrial proteins (MFN2, TFAM, NRF1), restore mitochondrial membrane potential, increase ATP production, and significantly reduce ROS accumulation, thereby ameliorating radiation-induced mitochondrial dysfunction in neurons.

### Exosomes regulate the differentiation potential of NSCs into astrocytes through the HMGB1/TLR2 pathway

**Fig. 5:**
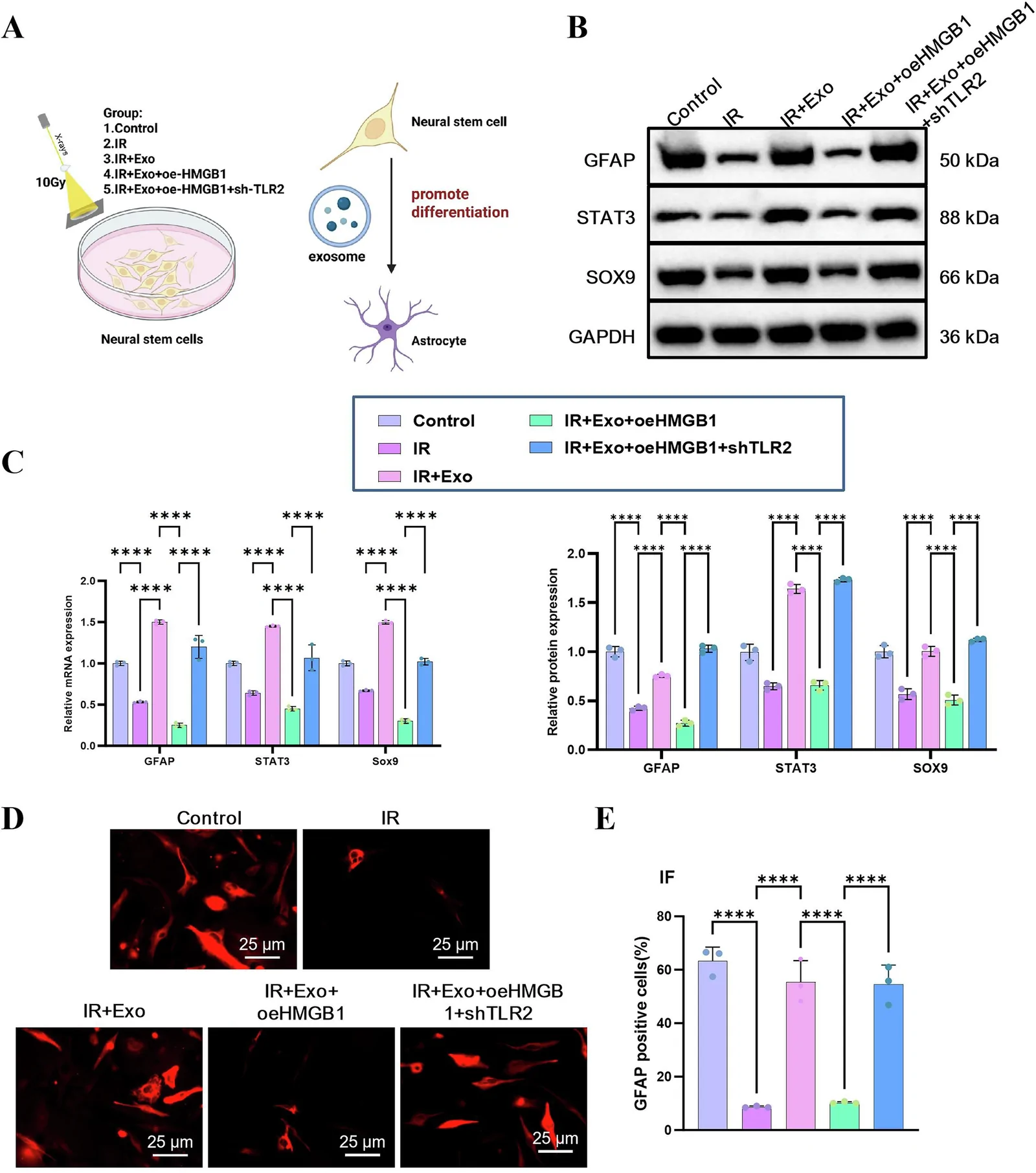
Effects of exogenous NSC-Exo on the differentiation of radiation-injured NSCs into astrocytes. **A** Schematic diagram of the experimental protocol investigating the effects of the HMGB1/TLR2 signaling pathway on the differentiation of NSCs into astrocytes; Western blot (**B**) and RT-PCR (**C**) analyses of GFAP, STAT3, and Sox9 protein expression and relative quantification in radiation-injured NSCs; **D**, **E** Immunofluorescence analysis of GFAP expression intensity in different treatment groups (bar = 25μm). Data are presented as mean ± standard deviation. *p < 0.05, **p < 0.01, ***p < 0.001, ****p < 0.0001.

In this part of the study, we investigated whether NSC-Exo promote the differentiation of NSCs into astrocytes by inhibiting the HMGB1/TLR2 signaling pathway (Fig. 5A).

WB and RT-qPCR analyses showed that, compared with the Control group, the IR group exhibited significantly reduced expression of the astrocyte marker GFAP and its upstream regulators STAT3 and SOX9. Exosome treatment restored the expression of these proteins, suggesting enhanced differentiation of NSCs toward an astrocytic lineage. HMGB1 overexpression attenuated this effect, whereas TLR2 knockdown promoted the upregulation of GFAP, STAT3, and SOX9 (Fig. 5B, C), indicating that exosomes facilitate astrocytic differentiation by modulating the HMGB1/TLR2 pathway.

Immunofluorescence staining further confirmed these findings. GFAP expression and fluorescence intensity were decreased in the IR group but markedly increased following exosome treatment, with cells displaying typical astrocytic morphology. HMGB1 overexpression suppressed GFAP expression, while TLR2 knockdown enhanced GFAP signals (Fig. 5D, E).

Flow cytometry was further used to quantify GFAP-positive cells. The IR group showed a significantly lower proportion of GFAP-positive cells than the Control group, indicating impaired astrocytic differentiation of NSCs after radiation injury. Exosome treatment increased the GFAP-positive cell population and restored differentiation capacity, whereas HMGB1 overexpression weakened this effect. In contrast, TLR2 knockdown rescued the exosome-mediated promotion of astrocytic differentiation (Fig. S5A, B). These results suggest that NSC-Exo may promote the differentiation of NSCs into astrocytes by suppressing activation of the HMGB1/TLR2 axis.

Altogether, these data demonstrate that exogenous NSC-Exos promote the differentiation of NSCs into astrocytes by inhibiting the HMGB1/TLR2 signaling pathway.

### The role of endogenous NSC differentiation into astrocytes in improving BBB function

**Fig. 6:**
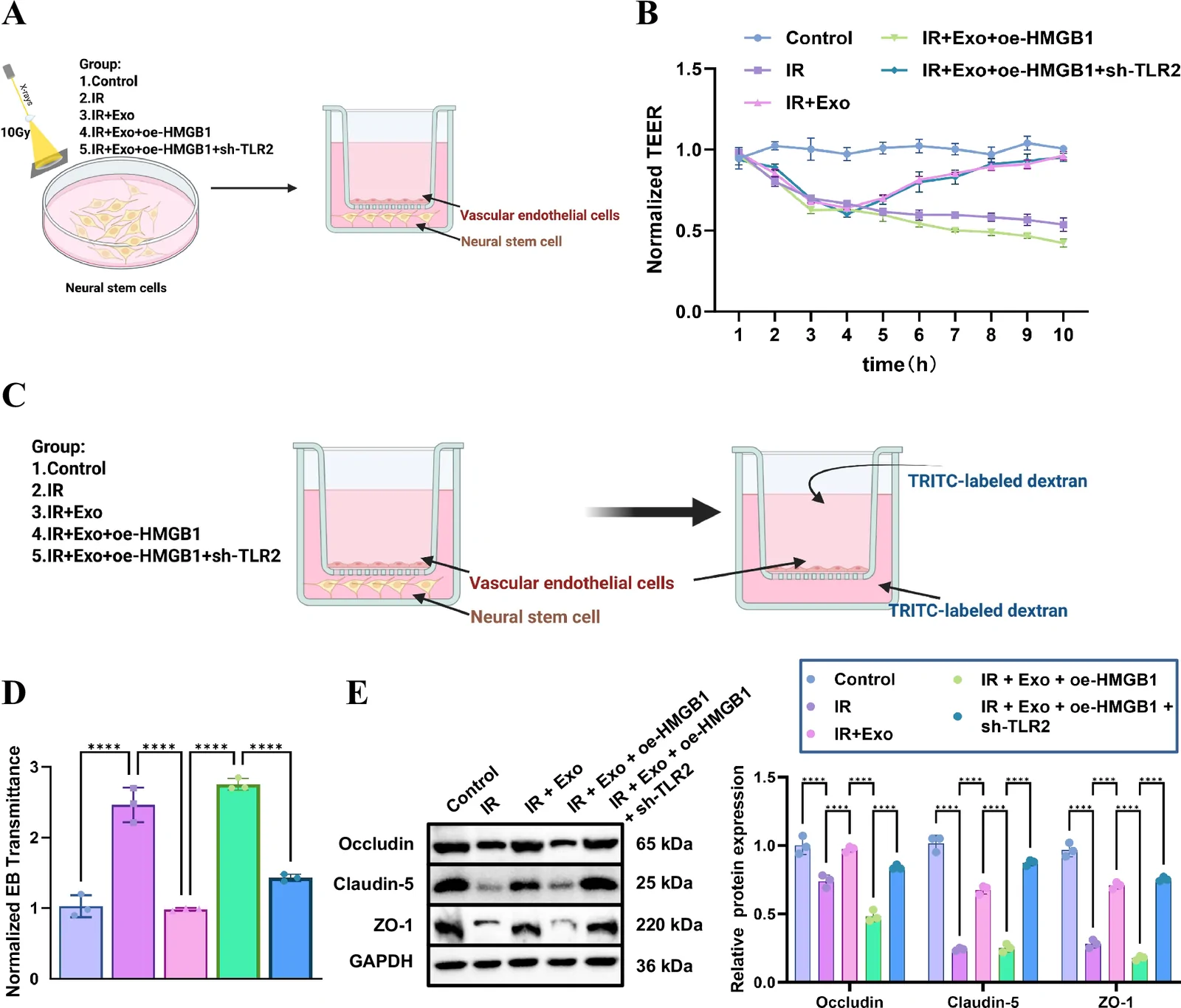
Effect of endogenous NSC differentiation into astrocytes on BBB function in a co-culture system. **A** Schematic diagram of the co-culture experimental workflow, illustrating the co-culture system of endogenous NSCs and BCECs under different treatment conditions; **B** TEER measurement of changes in electrical resistance across the BBB in the co-culture system to evaluate barrier function in each group; **C** Transwell schematic diagram showing TRITC-dextran leakage for assessing permeability after different treatments; **D** Evans blue permeability assay to assess BBB permeability in the co-culture system; **E** Western blot analysis and relative quantification of tight junction proteins Occludin, Claudin-5, and ZO-1 in the BBB. All cell experiments were repeated three times. Data are presented as mean ± standard deviation. **p < 0.01, ***p < 0.001, ****p < 0.0001.

In this experiment, building upon previous findings, we further investigated the effect of NSC-derived astrocytic differentiation on BBB-associated barrier function. A co-culture system of NSCs and rat BCECs was established (Fig. 6A).

TEER measurements were first performed to evaluate endothelial barrier integrity. Compared with the Control group, the IR group showed a significant decrease in TEER values, indicating barrier disruption after radiation. In contrast, exosome treatment significantly increased TEER values, suggesting improved barrier function. HMGB1 overexpression further reduced TEER, whereas TLR2 knockdown restored resistance levels (Fig. 6B), indicating that the HMGB1/TLR2 axis participates in exosome-mediated protection of BBB integrity.

Subsequently, the TRITC-dextran permeability assay further validated changes in endothelial permeability (Fig. 6C). The IR group exhibited significantly increased TRITC-dextran leakage compared to the Control group, indicating abnormal enhancement of barrier permeability. In the Exo group, TRITC-dextran leakage was markedly reduced, demonstrating the efficacy of exosome treatment in decreasing endothelial permeability. Overexpression of HMGB1 counteracted the protective effect of exosomes, while TLR2 knockdown again reduced permeability (Fig. 6D), further confirming that exosomes maintain barrier function by downregulating the HMGB1/TLR2 pathway.

WB analysis was performed to evaluate the expression of key tight junction proteins of the BBB, including Occludin, Claudin-5, and ZO-1. The expression of these proteins was significantly reduced in the IR group, whereas exosome treatment markedly increased their levels. HMGB1 overexpression suppressed this upregulation, while TLR2 knockdown partially restored protein expression (Fig. 6E), further indicating that BBB protection is mediated through the HMGB1/TLR2 signaling axis.

Thus, exosome intervention can improve BBB-associated barrier function after radiation injury by inhibiting the HMGB1/TLR2 signaling pathway, enhancing tight junction protein expression, reducing endothelial permeability, and increasing barrier resistance.

### NSC-Exo ameliorates inflammation and mitochondrial dysfunction after RBI by regulating the HMGB1/TLR2 signaling axis

**Fig. 7:**
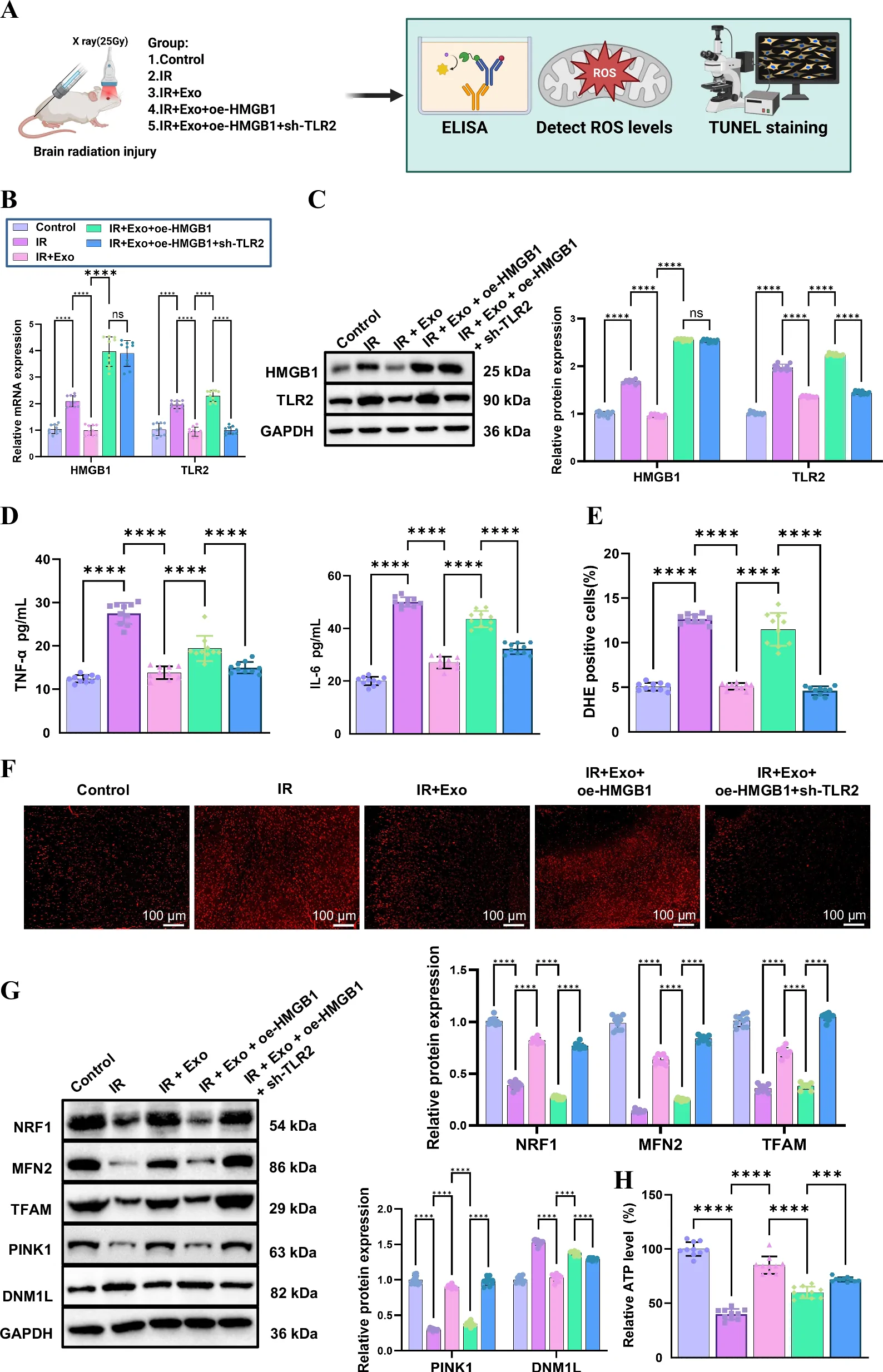
NSC-Exo improves the microenvironment and promotes mitochondrial biogenesis in RBI by inhibiting the HMGB1/TLR2 signaling pathway. **A** Schematic diagram of experimental protocols for different groups; qRT-PCR (**B**) and Western blot (**C**) analyses of HMGB1 and TLR2 expression in each group; **D** ELISA measurement of inflammatory cytokines TNF-α and IL-6 in brain tissue; **E**, **F** DHE staining to detect ROS levels in brain tissue (bar = 100 μm); **G** Western blot analysis of mitochondrial biogenesis-related protein expression in each group; **H** ATP colorimetric assay to measure ATP content in brain tissue, reflecting mitochondrial function. Each group included 10 rats. Data are presented as mean ± standard deviation. ***p < 0.001, ****p < 0.0001.

We established a rat model of RBI to systematically investigate whether NSC-Exo can alleviate radiation-induced neuroinflammation, oxidative stress, and mitochondrial dysfunction by modulating the HMGB1/TLR2 signaling pathway (Fig. 7A).

RT-qPCR and WB analyses were performed to assess the expression of key inflammatory factors, HMGB1 and TLR2, in brain tissue. Both proteins were significantly upregulated in the IR group, indicating activation of the inflammatory pathway following radiation. Exosome treatment markedly reduced their expression. In contrast, HMGB1 overexpression reversed this effect and restored elevated HMGB1 and TLR2 levels. TLR2 knockdown decreased TLR2 expression without significantly affecting HMGB1, suggesting that HMGB1 acts upstream to regulate TLR2 (Fig. 7B, C).

ELISA was used to measure TNF-α and IL-6 levels in brain tissue. The results showed that both cytokines were significantly elevated in the IR group compared to controls, indicating a robust inflammatory response after radiation injury. Exosome treatment significantly decreased TNF-α and IL-6 levels, demonstrating a pronounced anti-inflammatory effect. However, overexpression of HMGB1 again elevated inflammation, while combined TLR2 knockdown partially restored the anti-inflammatory effect, indicating that this effect is mediated through HMGB1/TLR2 signaling (Fig. 7D).

DHE staining showed that ROS levels in brain tissue were significantly elevated in the IR group, whereas exosome treatment markedly reduced ROS accumulation, indicating attenuation of oxidative stress. HMGB1 overexpression increased ROS levels, while TLR2 knockdown decreased ROS production (Fig. 7E, F), further confirming the involvement of the HMGB1/TLR2 axis in ROS regulation.

To further assess mitochondrial function, WB analysis was performed for mitochondrial-related proteins. In the IR group, the expression of NRF1, MFN2, TFAM, and PINK1 was decreased, while DNM1L was significantly increased, indicating impaired mitochondrial biogenesis and fusion with enhanced fission. Exosome intervention reversed these changes by increasing the expression of mitochondrial protective proteins and reducing the level of the fission protein DNM1L. Overexpression of HMGB1 significantly weakened the regulatory effects of exosomes, whereas TLR2 knockdown partially restored mitochondrial homeostasis (Fig. 7G).

Additionally, ATP colorimetric assay results showed that ATP levels were significantly decreased in the IR group and markedly restored in the Exo group. ATP production remained limited in the HMGB1 overexpression group, while TLR2 knockdown partially recovered ATP levels (Fig. 7H), further indicating that the restorative effect of exosomes on energy metabolism is dependent on HMGB1/TLR2 regulation.

Collectively, NSC-Exo can significantly improve inflammation, oxidative stress, and mitochondrial dysfunction induced by radiation brain injury in vivo. By enhancing the expression of fusion proteins, downregulating fission-related factors, and restoring energy metabolism, exosomes provide comprehensive neuroprotection.

### NSC-Exo attenuates BBB disruption after RBI by regulating the HMGB1/TLR2 pathway

**Fig. 8:**
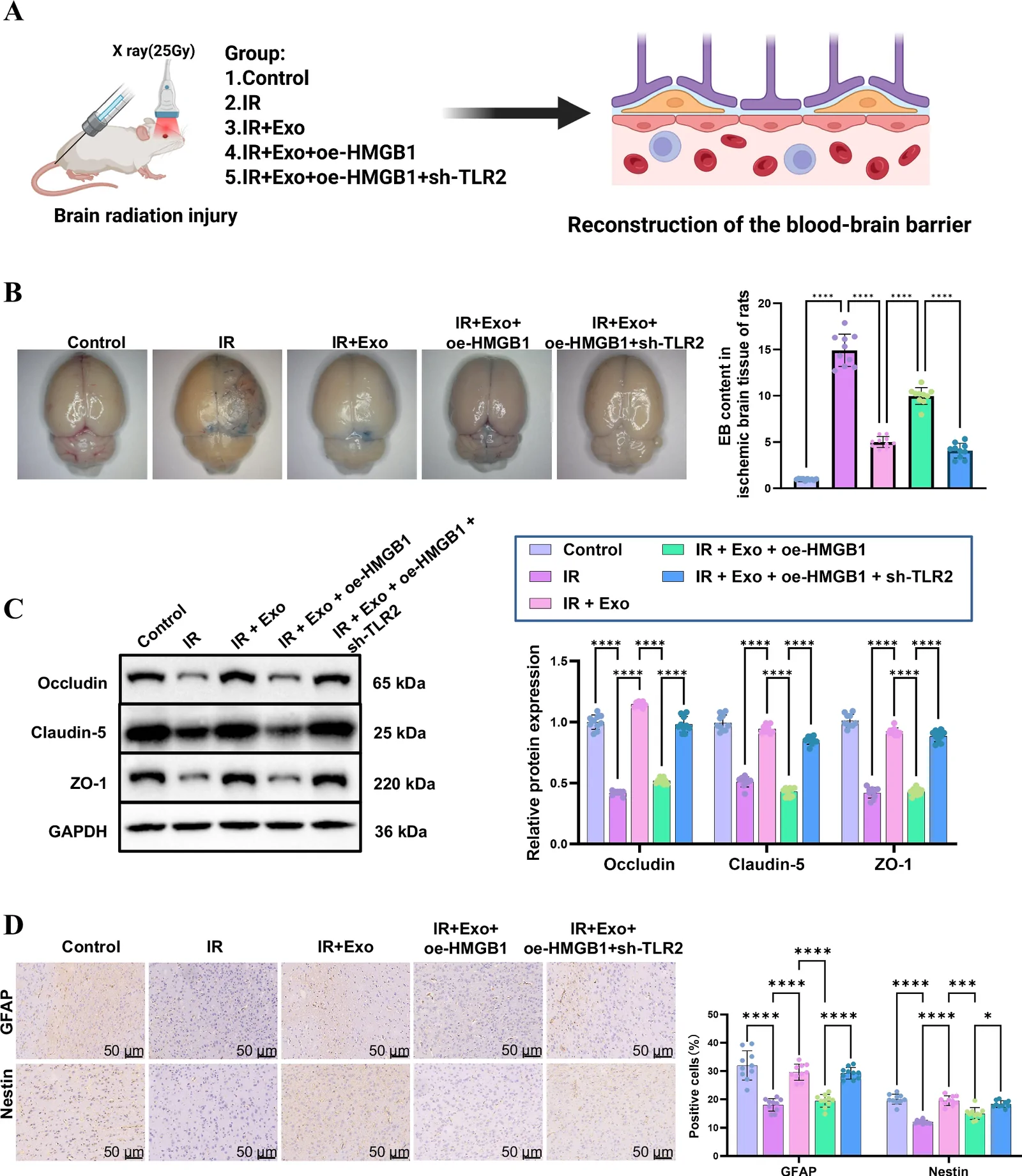
NSC-Exo promotes BBB remodeling after RBI via the HMGB1/TLR2 pathway. **A** Schematic diagram of the experimental workflow; **B** Evans blue permeability assay to assess BBB permeability in each group, presented as EB content (bar = 5 mm); **C** Western blot analysis of tight junction protein (Claudin-5, Occludin, ZO-1) expression; **D** Immunohistochemical staining for GFAP and Nestin to evaluate astrocyte differentiation and NSC marker expression. Each group included 10 rats. Data are presented as mean ± standard deviation. ****p < 0.0001.

In this part of the study, we investigated whether NSC-Exo attenuates BBB disruption after RBI in rats by regulating the HMGB1/TLR2 signaling pathway (Fig. 8A).

Evans blue permeability assays were performed to evaluate BBB integrity. Compared with the Control group, the IR group exhibited increased Evans blue extravasation into brain tissue, indicating enhanced BBB permeability. In contrast, exosome treatment markedly reduced dye leakage, suggesting improved barrier integrity. HMGB1 overexpression aggravated permeability, whereas TLR2 knockdown restored barrier function (Fig. 8B), highlighting the involvement of the HMGB1/TLR2 pathway in BBB protection.

At the protein level, WB analysis showed that the expression of key BBB tight junction proteins Claudin-5, Occludin, and ZO-1 was significantly downregulated in the IR group. Exosome intervention markedly increased the expression of all three proteins, suggesting that exosomes enhance BBB stability by upregulating tight junction proteins. HMGB1 overexpression attenuated these improvements, while TLR2 knockdown restored protein levels (Fig. 8C), confirming that this effect is regulated by the HMGB1/TLR2 axis.

Finally, immunohistochemical staining showed that GFAP-positive and Nestin-positive signals were reduced in the IR group, indicating impaired astrocytic marker expression and NSC-associated marker expression after RBI. After exosome intervention, GFAP expression increased significantly, and the number of Nestin-positive cells also notably recovered, suggesting improved astrocytic differentiation-related signals and partial recovery of NSC-associated stemness markers, thereby providing microenvironmental support for BBB protection (Fig. 8D).

Taken together, these results demonstrate that exosomes derived from NSCs can attenuate BBB disruption and improve barrier-associated function after RBI. The underlying mechanisms may include inhibition of the HMGB1/TLR2 signaling pathway, promotion of NSC differentiation into astrocytes, and enhancement of tight junction protein expression.

## Discussion

This study systematically elucidates the mechanism by which exogenous NSC-Exo improves BBB-associated function after RBI through regulation of the HMGB1/TLR2 signaling axis, enhancement of endogenous NSC mitochondrial biogenesis, and promotion of differentiation into astrocytes. Building on existing neural stem cell therapy concepts, this research employed a cell-free therapeutic strategy, expanding potential avenues for neural tissue repair. The study clarifies the neuroprotective effects of NSC-Exo and reveals multiple mechanisms by which these exosomes regulate specific molecular signaling axes to remodel cellular energy metabolism and differentiation fate, providing theoretical support for the treatment of complex brain injuries. By systematically validating these mechanisms in the context of RBI, this work extends the current understanding of exosome-mediated repair and offers potential significance for both academic research and clinical translation.

Previous studies have shown that exosomes can modulate inflammation and promote neuroregeneration in traumatic brain injury and other neurological insults [35, 36]. The present study extends this knowledge to RBI and focuses on BBB-associated functional improvement after radiation exposure. Through systematic evaluation using Evans blue permeability, TEER measurement, TRITC-dextran leakage, and tight junction protein analysis, we found that NSC-Exo intervention reduced BBB permeability and improved barrier-associated integrity. Furthermore, the study explored the underlying mechanisms, showing that NSC-Exo not only maintains NSC-related stemness markers but also promotes their differentiation toward an astrocytic lineage. These findings enrich the understanding of exosome-mediated neural repair at the levels of cells, the microenvironment, and the BBB.

The inflammatory regulatory role of the HMGB1/TLR2 signaling axis has been confirmed in various neurological disorders such as stroke and traumatic brain injury, with many studies focusing on its mediation of inflammation and BBB disruption [37, 38]. Our results demonstrate that NSC-Exo effectively suppresses the expression of HMGB1 and downstream TLR2, reduces ROS levels, and improves mitochondrial function and energy metabolism. Unlike previous approaches that relied mainly on inhibitors or genetic knockout for direct pathway intervention, this study achieves indirect and flexible regulation of HMGB1/TLR2 via exosomes. Molecular intervention experiments revealed that HMGB1 overexpression attenuates the protective effects of NSC-Exo, whereas TLR2 knockdown partially restores these effects, further verifying the key role of this signaling axis in NSC-Exo-mediated brain injury repair and providing new ideas for molecular targeted therapy of related diseases.

HMGB1-mediated neuroinflammatory signaling involves TLR2, TLR4, and RAGE-dependent pathways. These receptor cascades are associated with cytokine release, microglial activation, endothelial stress, and BBB permeability. The present findings identify HMGB1/TLR2 signaling as a key pathway through which NSC-Exo modulates inflammatory activation, mitochondrial dysfunction, and BBB-associated injury after RBI, within this broader HMGB1-related inflammatory network [27, 28].

Mitochondrial dysfunction is a major limiting factor in neural repair, and previous studies have demonstrated that mitochondrial impairment affects energy metabolism, exacerbates oxidative stress, and promotes cell apoptosis [39, 40]. The maintenance of stemness and differentiation of NSCs is highly dependent on mitochondrial function [29–31]. In this study, we systematically evaluated the effects of NSC-Exo on mitochondrial function using JC-1 staining, ATP measurement, and DHE staining. The results showed that exosome intervention significantly increased mitochondrial membrane potential and ATP levels, reduced ROS production, and improved the energy metabolic status of endogenous NSCs. These results corroborate the established theory that mitochondrial function determines NSC fate [30]. Furthermore, consistent with previous reports that exosomes support mitochondrial homeostasis in various injury models, our findings highlight mitochondrial biogenesis as a key target of NSC-Exos in RBI. These results underscore the importance of metabolic reprogramming in neural repair and provide a theoretical basis for developing metabolism-targeted therapeutic strategies.

Extracellular vesicles may also regulate mitochondrial homeostasis through cargo-dependent mechanisms that extend beyond suppression of inflammatory signaling. NSC-derived vesicles have been reported to carry mitochondrial proteins and to support horizontal transfer of functional mitochondria or mitochondrial components, providing a plausible route by which vesicles could stabilize bioenergetic function in injured neural cells [19, 20]. In the present dataset, the restoration of TFAM, MFN2, NRF1, ATP production, and mitochondrial membrane potential is therefore consistent with both inflammatory pathway inhibition and broader vesicle-mediated metabolic support.

Astrocytes play an essential role not only in neuronal support and regulation of neuroinflammation but are also indispensable for the maintenance of BBB structure and function [41]. Numerous studies have reported that astrocyte dysfunction can directly lead to BBB disruption and imbalance in the neural microenvironment [41–43]. Our results demonstrated that NSC-Exo treatment enhanced GFAP expression and increased the proportion of GFAP-positive cells in the NSC differentiation model, indicating that exosome intervention promotes NSC differentiation toward an astrocytic lineage. In parallel, in vivo data showed improved tight junction protein expression and reduced Evans blue leakage after NSC-Exo treatment. These findings suggest that astrocytic differentiation-related changes may contribute to a supportive microenvironment for BBB protection. Unlike strategies that primarily focus on BBB protection through immunosuppression or pharmacological approaches, this study provides a molecular and cellular perspective involving stem cell-derived exosomes, inflammatory signaling, mitochondrial function, and astrocytic differentiation.

At the same time, the relationship between astrocytic differentiation and BBB-associated functional improvement should be interpreted as supportive rather than definitively causal. Astrocytes are required for adult BBB maintenance and can regulate endothelial permeability through soluble factors and inflammatory signaling [44, 45]. The parallel increase in GFAP-positive cells and improvement in TEER, dextran permeability, and Evans blue leakage suggests astrocyte-associated support of barrier protection, but NSC-Exo may also act directly on endothelial cells, microglia, or pericyte-associated pathways. Future blockade of STAT3/SOX9-dependent astrocytic differentiation would be needed to separate direct anti-inflammatory protection from indirect astrocyte-mediated barrier support.

This study systematically and comprehensively analyzed the regulatory effects and underlying mechanisms of NSC-Exo through the integration of transcriptomics and molecular and cellular biology experiments. Unlike previous studies that mostly relied on the detection of single proteins or signaling molecules, our transcriptomic analysis not only revealed the enrichment of key pathways such as mitochondrial biogenesis and immune regulation, but also provided a data foundation for subsequent molecular screening and mechanistic investigation. The qPCR validation of additional targets such as TNF and MT1 further supported the findings of the transcriptomic analysis. The integration of multi-omics analysis with molecular intervention significantly enhanced the systematic nature and persuasiveness of the study, contributing to the establishment of a more comprehensive theoretical framework for brain injury repair. Especially given the complex action network and widespread targets of exosomes, our multifaceted and systematic approach stands out and offers a valuable model for future research in related fields.

Transcriptomic screening also highlighted additional candidates, including TNF and MT1, that may reflect broader immunometabolic effects of NSC-Exo beyond HMGB1/TLR2. Their qPCR validation supports the RNA-seq trends and reinforces the view that exosome treatment acts through a network of inflammatory, oxidative, and metabolic regulators rather than a single linear pathway.

In terms of functional validation of signaling pathways, this study used molecular intervention strategies such as HMGB1 overexpression and TLR2 knockdown to clarify the critical role of the HMGB1/TLR2 axis in NSC-Exo-mediated brain injury repair. This strategy not only addressed the limitation of correlation-based analyses seen in related research, but also established a causal relationship between exosome action, signaling axis, and functional outcomes through functional experiments. Compared with traditional pharmacological inhibitors or animal gene knockout models, molecular intervention experiments offer flexibility, strong target specificity, and direct mechanistic insights, which facilitate the development of precise intervention strategies. Additionally, the molecular intervention approach used in this study is of great significance for both mechanistic research and translational medicine, and advances the field of stem cell-derived exosome research.

Candidate vesicular cargos remain an important mechanistic layer. Exosomal miR-129-5p has been linked to HMGB1 suppression in inflammatory injury contexts, and NSC-derived vesicles may carry mitochondrial or RNA cargos capable of shaping recipient-cell metabolism [20, 46]. Defining the active miRNAs, proteins, and organelle-related cargos in NSC-Exo will be necessary to move from pathway-level association toward cargo-level mechanism.

In summary, this study systematically demonstrates that NSC-Exos can enhance endogenous NSC mitochondrial biogenesis and differentiation capacity, and collaboratively repair the BBB after RBI by suppressing the HMGB1/TLR2 signaling axis. This finding enriches the theoretical framework of RBI and BBB repair, and provides new targets and intervention strategies for the precise treatment of neurological injuries. However, there are still limitations to this study.

Several aspects limit the generalizability of these findings. The in vivo experiments used young male SD rats and an acute single-dose irradiation model, whereas clinical radiation injury often develops after fractionated exposure and can progress over months to years with chronic vascular remodeling, demyelination, and late radionecrosis. Sex-dependent differences in BBB transport and neuroinflammatory responses may also affect therapeutic responses [47]. Long-term studies in both sexes and in clinically relevant fractionated irradiation models will be required to determine whether the short-term improvements in cognition and BBB function persist and whether NSC-Exo can prevent delayed tissue injury.

## Conclusion

This study demonstrates that NSC-Exo improve BBB-associated integrity after RBI by inhibiting the HMGB1/TLR2 signaling pathway, promoting mitochondrial biogenesis, reducing oxidative stress and apoptosis, and enhancing the differentiation of NSCs into astrocytes. These collective effects support the multiple protective roles of NSC-Exo in improving the neural microenvironment and facilitating tissue repair.

Our findings reveal the potential of NSC-Exo to regulate RBI-associated barrier dysfunction via the HMGB1/TLR2 pathway, highlighting the therapeutic value of exosome-based treatments for neuroprotection and BBB protection. This mechanism offers a new perspective for the development of exosome-based intervention strategies for RBI, with potential clinical relevance for the prevention and recovery of neurological complications arising from tumor radiotherapy.

Despite uncovering the important role of exosomes in brain injury repair, this study has certain limitations. Firstly, the research was conducted primarily in animal models, and future studies will require validation in clinical samples to ensure safety and efficacy in humans. In addition, this study mainly explored the regulatory role of the HMGB1/TLR2 signaling pathway; further investigations are warranted to elucidate other potential molecular mechanisms. Exosomes hold great promise for drug delivery systems and neuroprotection, and in the future, engineering approaches may enhance their targeting and therapeutic efficiency, thereby advancing the development of precision medicine.

## Materials and methods

### Exosome isolation and identification

NSC-Exos were isolated by ultracentrifugation. Briefly, culture supernatants were sequentially centrifuged at 3000 × g for 10 min and 10,000 × g for 30 min to remove cells, debris, and large particles. Exosomes were then collected by ultracentrifugation at 100,000 × g for 2 h and resuspended in PBS. Their morphology was examined by transmission electron microscopy (TEM; JEOL, Japan), particle size was measured using nanoparticle tracking analysis (NTA; Malvern, UK), and exosomal markers, including Alix, Hsp90, CD63, and Tsg101, were detected by WB.

### Western blot

Total protein was extracted from cells and tissues. Cells were collected after trypsin digestion (T4799-5G, Sigma-Aldrich, USA), and tissue samples were processed similarly. All samples were lysed with enhanced RIPA lysis buffer containing protease inhibitors (AR0108, BOSTER, Wuhan). Protein concentrations were determined using a BCA protein quantification kit (AR1189, BOSTER). Equal amounts of protein were separated by SDS-PAGE and transferred onto PVDF membranes. After blocking with 5% BSA (9048-46-8, Sigma-Aldrich, USA) for 1 h at ambient temperature, membranes were incubated with primary antibodies (Table S1) overnight at 4 °C. After three washes with PBST, membranes were incubated with HRP-conjugated anti-mouse (Cat# 7076, 1:5000, Cell Signaling Technology, USA) or anti-rabbit secondary antibody (Cat# 7074, 1:5000, Cell Signaling Technology, USA) for 1 h at ambient temperature. Protein signals were visualized using enhanced chemiluminescence substrate (Omt-01, OMIGET, Beijing), exposed to X-ray film in the dark for 5–10 min, and analyzed with ImageJ. GAPDH was used as the internal control. Full and uncropped western blot images are provided in the Supplemental Material.

### Animal experiment design and grouping

Male SPF Sprague-Dawley rats aged 6–8 weeks and weighing 210–260 g were obtained from the Experimental Animal Center. After one week of acclimatization, rats were housed under controlled temperature and humidity with a 12 h light/dark cycle (lights on 7:00–19:00) and free access to food and water.

For irradiation, rats in all groups, including controls, were anesthetized by intraperitoneal injection of sodium pentobarbital (50 mg/kg). Animals were placed prone with the dorsal head facing the linear accelerator. Except for controls, rats received frontal-lobe X-ray irradiation using an Elekta Synergy linear accelerator (6 MeV, UK) at 300 MU/min, with a total dose of 25 Gy [48].

For exosome intervention, NSC-Exos were administered via tail vein immediately after model establishment on the irradiation day at 100 μg/kg, followed by injections every other day for two weeks. At the end of the experiment, rats were euthanized by cervical dislocation under deep isoflurane anesthesia (R510-22-10, RWD Life Science, Shenzhen, China). All procedures were approved by the Animal Ethics Committee of the local hospital and conducted in accordance with animal experimental guidelines (Ethics Approval Number: 20240566) [49].

Rats were randomly assigned to five groups (n = 10/group): Control; IR (radiation injury + oe-NC + sh-NC); IR+Exo (radiation injury + NSC-Exos + oe-NC + sh-NC); IR+Exo+oeHMGB1 (radiation injury + NSC-Exos + oeHMGB1 + sh-NC); and IR+Exo+oeHMGB1+shTLR2 (radiation injury + NSC-Exos + oeHMGB1 + shTLR2).

### Cell culture and grouping

Primary rat NSCs (PC-145r), hippocampal neurons (HNCs, PC-112r), and brain capillary endothelial cells (BCECs, CL-057r) were purchased from Saios Biotechnology Co., Ltd. (Wuhan, China) and cultured under standard conditions. Cells were maintained in DMEM/F12 medium supplemented with 10% fetal bovine serum (FBS; Gibco, USA), 1% penicillin–streptomycin (Thermo Fisher Scientific, USA), and 2% B27 supplement (Gibco, USA), and incubated at 37 °C in a humidified atmosphere with 5% CO_2_.

Cells were divided into five groups: Normal control group (Control); Radiation injury control group (IR; cells exposed to 10 Gy X-ray irradiation, radiation injury plus injection of oe-NC and sh-NC); Exosome intervention group (IR+Exo; radiation injury plus exosome intervention, exosome concentration 50 μg/ml, plus injection of oe-NC and sh-NC); Exosome plus HMGB1 overexpression group (IR+Exo+oeHMGB1; radiation injury plus exosome intervention, plus oeHMGB1 and sh-NC); Exosome plus HMGB1 overexpression and TLR2 knockdown group (IR+Exo+oeHMGB1+shTLR2; radiation injury plus exosome intervention, plus oeHMGB1 and shTLR2) [49–51].

### Uptake of fluorescently labeled exosomes by recipient cells

Exosomes were labeled using an Exo-Green exosome protein labeling kit (EXOG200A-1, System Biosciences, USA). Briefly, 50 μL of 10× Exo-Green was added to 500 μL exosome suspension in 1× PBS and gently mixed, followed by incubation at 37 °C for 10 min. FBS (A5669701, Thermo Fisher, USA) was then added to terminate the reaction. The labeled exosomes were kept at 4 °C for 30 min and centrifuged at 14,000 rpm for 3 min. After removing the supernatant containing excess dye, the exosomes were resuspended in PBS for subsequent use.

A total of 8 μg labeled exosomes were added to recipient cell culture medium and incubated at 37 °C for 3 h. Cells were then fixed with 4% paraformaldehyde at ambient temperature for 10 min, permeabilized with 0.1% Triton X-100 for 5 min, and stained with DAPI (D1306, Thermo Fisher, USA). After mounting, cells were observed under a fluorescence microscope.

### Cell viability assay

NSCs and HNCs were seeded in 96-well plates at 1 × 10^4^ cells/well and cultured for 24 h. Cells in each treatment group were then incubated with 0.5 mg/mL MTT (298-93-1, Sigma-Aldrich, USA) for 4 h. After removing the MTT solution, 100 μL dimethyl sulfoxide (DMSO; 67-68-5, Sigma-Aldrich, USA) or another suitable solvent was added to dissolve the formazan crystals. Cell viability was evaluated by measuring absorbance at 570 nm using a microplate reader.

### In vivo exosome tracking

Rats from different treatment groups were randomly selected for in vivo imaging. After a single administration of labeled exosomes, animals were anesthetized with isoflurane according to body weight. Fluorescence images were acquired at 35, 150, 240, and 360 min using an IVIS Lumina XR system with a laser power of 70–80 V. Fluorescence imaging was performed with excitation at 488 nm and emission at 550–650 nm.

### Immunofluorescence staining

Cells and tissue sections were rinsed with ice-cold PBS, fixed in 4% paraformaldehyde (P885233, Macklin, USA) for 15–30 min, and permeabilized with 0.1% Triton X-100 (L885651, Macklin, USA) for 15 min. After PBS rinsing, nonspecific binding was blocked with 15% FBS in PBS at 5 °C for 4 min. Samples were then incubated overnight at 4 °C with rabbit anti-CD11b (ab7260, Abcam, USA; 1:100), rabbit anti-CD31 (ab222783, Abcam, USA; 1:100), or mouse anti-VEGF (MA5-13182, Thermo Fisher, USA; 1:100). After TBST washing, samples were labeled for 2 h at ambient temperature with goat anti-rabbit Alexa Fluor® 647 (A-21245, Thermo Fisher, USA) or goat anti-mouse Alexa Fluor Plus 488 (A32723, Thermo Fisher, USA), followed by nuclear staining with DAPI (D1306, Thermo Fisher, USA). Images were obtained using a Zeiss Observer Z1 microscope (Germany). Fluorescence intensity within selected regions and positive-cell counts were quantified with ImageJ.

### Assessment of BBB permeability (Evans blue assay)

To evaluate the effect of exosomes on BBB permeability, rats received an intravenous injection of 2% Evans blue dye (4 mL/kg; E2129, Sigma-Aldrich, USA), with the dose adjusted according to body weight after model establishment. Following injection, the rats were allowed to move freely for 30 min to ensure sufficient distribution of the dye throughout the circulation. The rats were then anesthetized with sodium pentobarbital (50 mg/kg) and transcardially perfused with ice-cold physiological saline until the outflow became clear to remove intravascular dye. The brains were rapidly removed, and the same anatomical region was collected from each animal for analysis. Brain tissue was weighed and cut into small pieces, which were then placed into centrifuge tubes containing formamide (F0507, Sigma-Aldrich, USA) at a ratio of 1 mL formamide per gram of brain tissue, ensuring complete immersion. The tubes were incubated in a 55 °C water bath for 24 h to extract Evans blue dye thoroughly. After incubation, the samples were centrifuged at 12,000 rpm for 15 min, and the supernatant was collected. The absorbance of the supernatant was measured at 620 nm using a spectrophotometer (Thermo Fisher Scientific, Model: Genesys 10S UV-Vis). The content of Evans blue in each sample was calculated based on a standard curve using the following formula: Evans blue content (μg/g) = (absorbance value/standard curve slope) / brain tissue weight (g). The Evans blue content of each group was compared to analyze the effect of exosomes on BBB permeability.

### Tissue TUNEL assay

Fixed tissues were dehydrated, paraffin-embedded, and sectioned at 3 μm thickness. Apoptosis was assessed using a TUNEL apoptosis detection kit (ab66110, Abcam, USA). Briefly, sections were incubated with TUNEL reaction mixture at 37 °C in a humidified dark chamber for 60 min, followed by PBS washes to remove excess reagents.

Apoptotic cells were first observed under a microscope at 100× magnification. For quantification, three randomly selected non-overlapping fields per section were analyzed at 200× magnification to determine the number of TUNEL-positive cells relative to total cells, and the mean value was calculated. Typical apoptotic features included reduced cell size, chromatin condensation, and apoptotic body formation.

### RNA extraction and sequencing

Brain tissues from rats in the IR and exosome (Exo) groups were collected (n = 3 per group). Total RNA was extracted using TRIzol reagent (15596026, Invitrogen, USA), and RNA concentration and purity were assessed with a NanoDrop 2000 spectrophotometer (1011U, NanoDrop, USA). Library construction and sequencing were performed by CapitalBio Technology (Beijing, China), using 5 μg RNA per sample.

Ribosomal RNA was removed with the Ribo-Zero™ Magnetic Kit (MRZE706, Epicentre Technologies), and sequencing libraries were prepared using the NEBNext Ultra RNA Library Prep Kit (E7775, NEB, USA) for Illumina platforms. RNA was fragmented in NEBNext First Strand Synthesis Reaction Buffer (5×) to ~300 bp. First-strand cDNA was synthesized using reverse transcriptase and random primers, followed by second-strand synthesis in a buffer containing dUTP Mix (10×). The cDNA fragments were end-repaired, A-tailed, and ligated to sequencing adapters. After adapter ligation, USER Enzyme (#M5508, NEB, USA) was applied to digest the second strand and generate strand-specific libraries. Libraries were then amplified, purified, and enriched by PCR. Quality was evaluated using the Agilent 2100 system, and quantification was performed with the KAPA Library Quantification Kit (KK4844, KAPA Biosystems). Finally, paired-end sequencing was conducted on an Illumina NextSeqCN500 platform.

### Sequencing data processing and alignment

Raw paired-end reads were evaluated using FastQC (v0.11.8). Adapter sequences and poly(A) tails were removed with Cutadapt (v1.18). Reads containing >5% ambiguous bases (N) were discarded, and reads with ≥70% bases having a quality score >20 were retained using the FASTX Toolkit (v0.0.13). Error correction of paired-end reads was performed with BBMap. Clean, high-quality reads were subsequently aligned to the rat reference genome using HISAT2 (v0.7.12).

### Differential expression analysis

Gene expression data were normalized and quantile-corrected using the Limma package (v3.48.3) in R to reduce batch effects and technical variation. Differentially expressed genes (DEGs) were identified with DESeq2 (v1.32.0), using thresholds of *p* < 0.05 and |log_2_FC | > 1.

### Gene ontology (GO) and Kyoto Encyclopedia of Genes and Genomes (KEGG) enrichment analysis

GO and KEGG enrichment analyses were conducted using the “ClusterProfiler” package in R, with *p* < 0.05 as the significance threshold for enrichment. GO analysis included biological process (BP), molecular function (MF), and cellular component (CC) categories. The analyses identified the major cellular functions, signaling pathways, and disease-associated pathways in which the differentially expressed genes (DEGs) were enriched. The “ClusterProfiler” package was also used to visualize the KEGG enrichment results with bubble plots, based on *p* values.

### Least absolute shrinkage and selection operator (LASSO) regression algorithm

To identify key DEGs associated with exosome intervention, LASSO regression was applied for feature selection and dimensionality reduction. The glmnet package was implemented in R with a fixed random seed to ensure reproducibility. DEG expression matrices were used as input variables, and sample groups (IR vs. IR_Exo) were defined as the response variable. The model was constructed using the glmnet() function, and the optimal regularization parameter (λ) was determined via tenfold cross-validation using cv.glmnet(). Genes with nonzero coefficients at the optimal λ were selected as candidate genes related to exosome intervention.

### Correlation heatmap analysis

Pairwise correlations among variables were calculated using the Spearman method. Results were visualized as heatmaps with the R packages igraph (v1.4.1) and ggraph (v2.1.0). Correlation strength was represented by the coefficient value, where a larger absolute value indicates a stronger association.

### Protein–protein interaction (PPI) network construction

The STRING database (http://www.string-db.org/) was used to construct the PPI network, integrating evidence from experimental data, PubMed text mining, other databases, and computational predictions. Protein–protein interactions among candidate genes, including characteristic and mitochondria-related genes, were analyzed using STRING. The resulting network was visualized and further analyzed in Cytoscape software (v3.7.2). Node degree (number of connections) was calculated for each protein, with higher degree values indicating greater centrality and potential key regulatory roles within the network.

### Lentivirus preparation

The HMGB1 overexpression plasmid was constructed using the pCMV6-AC-GFP vector (LM-2069, LMAI Bio, Shanghai, China) and synthesized by Sangon Biotech (Shanghai, China). The pLKO.1-puro vector (QYV0024, QuaYad, Beijing, China) was used to generate rat TLR2-shRNA (5′-CACTTGGACTTGTCTAATAA-3′) and sh-NC (5′-TTCTCCGAACGTGTCACGT-3′), both purchased from Thermo Fisher (USA). Lentiviral vectors for HMGB1 overexpression (oeHMGB1), TLR2 knockdown (shTLR2), and their respective controls (oe-NC and sh-NC) were packaged in HEK293T cells (CBP60661, Cobioer, Jiangsu, China). Plasmid construction and viral packaging were performed by Sangon Biotech.

For transduction, 5 × 10^5^ cells were seeded per well in 6-well plates. Upon reaching 70–90% confluence, cells were infected with lentivirus-containing medium (MOI = 10; ~5 × 10^6^ TU/mL) supplemented with 5 μg/mL polybrene (TR-1003, Merck, USA). After 4 h, fresh medium was added to dilute polybrene, and the medium was replaced again after 24 h. Transduction efficiency was evaluated at 48 h using a luciferase reporter. Stable cell lines were selected with 10 μg/mL puromycin (A1113803, Gibco, USA). Cells were harvested once selection was complete, and overexpression or knockdown efficiency was confirmed by RT-qPCR.

### RT-qPCR

Total RNA was extracted from cells using the Trizol reagent kit (10296010, Invitrogen, Thermo Fisher, USA). The quality and concentration of RNA were measured by ultraviolet-visible spectrophotometry (ND-1000, Nanodrop, Thermo Fisher, USA). Reverse transcription was performed using either a poly(A) tailing kit (B532451, Sangon Biotech, Shanghai, China) or the PrimeScript™ RT-qPCR kit (RR086A, TaKaRa, Mountain View, CA, USA). qPCR was carried out using SYBR Premix Ex Taq™ (DRR820A, TaKaRa) on a LightCycler 480 system (Roche Diagnostics, USA). U6 served as the internal control for miRNA, while GAPDH was used for lncRNA and mRNA normalization. Primers were designed and synthesized by Shanghai Sangon Biotech, and sequences are listed in Table S2. Relative gene expression was calculated using the 2^-ΔΔCt^ method.

### Annexin V-FITC/propidium iodide (PI) staining

Cell apoptosis was assessed using the Annexin V-FITC/PI double staining kit (V13242, Thermo Fisher, USA) and analyzed by flow cytometry (CytoFLEX S, Beckman Coulter Inc., California, USA). Cells were seeded in 6-well plates at 5 × 10^5^ cells/well, allowed to adhere, and treated as indicated. Cells were then collected, washed twice with pre-chilled PBS, and resuspended in Annexin V binding buffer. Subsequently, 5 μL Annexin V-FITC (10 μg/mL) and 10 μL PI (20 μg/mL) were added, followed by incubation at ambient temperature in the dark for 20 min. Samples were immediately analyzed by flow cytometry. Apoptotic cell populations were identified based on FITC and PI fluorescence, and the percentage of apoptotic cells was calculated.

### Cell TUNEL Assay

Cell apoptosis was evaluated using a TUNEL assay kit (E-CK-A320, Wuhan Pricella Biotechnology Co., Ltd., China). Briefly, cells were seeded in 8-well chamber slides, washed with PBS, and fixed in 4% paraformaldehyde at ambient temperature for 1 h. After staining, nuclei were counterstained with DAPI to determine the total cell number. The apoptotic index (AI) was calculated as the percentage of TUNEL-positive cells among total cells (AI = TUNEL-positive cells/total cells × 100%).

### JC-1 Staining

Cells (2 × 10^4^) were seeded in 35 mm dishes and cultured overnight. After treatment for 24 h, cells were washed with PBS and incubated with JC-1 dye (T3168, Thermo Fisher, USA) at 37 °C for 20 min. Following washing, cells were mounted and observed under a fluorescence microscope. JC-1 aggregates (red fluorescence) and monomers (green fluorescence) were detected to assess mitochondrial membrane potential. Images from multiple random fields were captured and analyzed using ImageJ software. The ratio of red to green fluorescence intensity was calculated as an indicator of mitochondrial membrane potential.

### ATP assay

Mitochondrial function was evaluated by measuring ATP levels using an ATP colorimetric assay kit (ATP Assay Kit, 213-579-1, Thermo Fisher, USA). After experimental treatments, cells were lysed and the lysates were added to 96-well plates. ATP detection reagent was added according to the manufacturer’s instructions, followed by incubation at 37 °C for 15 min. Absorbance was then measured at 570 nm using a microplate reader (BioTek, USA).

### ROS Detection

Intracellular ROS levels were determined using a dihydroethidium (DHE) staining kit (104821-25-2, Biofount, Beijing, China). After treatment, cells were washed with PBS and incubated with DHE at 37 °C for 30 min. Fluorescence signals were then observed under a fluorescence microscope, and intensity was quantified to evaluate ROS levels.

### Flow cytometry

The anti-glial fibrillary acidic protein (GFAP) antibody (13-0300, Thermo Fisher, USA) was conjugated to a fluorescent dye using a PerCP-Cy5.5 labeling kit (ab102911, Abcam, USA). Treated cells were collected and prepared as single-cell suspensions. The resulting cells were fixed and permeabilized using the Cytofix/Cytoperm™ kit (BD Biosciences). The cells were then incubated with PerCP-Cy5.5-conjugated anti-GFAP antibody in the dark. After staining, samples were analyzed using a FACS Calibur flow cytometer (BD Biosciences). The proportion of GFAP-positive cells was determined to assess astrocytic differentiation.

### Transendothelial electrical resistance (TEER) measurement

TEER was used to evaluate the integrity of the BBB. BCECs were seeded into Transwell inserts in a co-culture system, and NSCs subjected to different treatments were added. TEER values were recorded using an EVOM epithelial voltmeter (World Precision Instruments, USA). Background resistance from blank inserts was subtracted, and values were normalized to baseline levels to assess changes in barrier function.

### Permeability assay of brain capillary endothelial cells (BECs)

ECs were seeded in the upper chamber of 24-well Transwell inserts (0.4 μm pore size, Costar, 11820050) at a density of 5 × 10^4^ cells/well and cultured for 72 h to form a confluent monolayer. Subsequently, complete DMEM or glucose-free DMEM (11966-025, Gibco) containing 2 mg/mL TRITC-dextran (4.4 kDa; T1037, Sigma-Aldrich) was added to the upper chamber. Medium samples (50 μL) were collected from both upper and lower chambers, and fluorescence intensity (RFU) was measured using a microplate reader (SpectraMax M5) at excitation/emission wavelengths of 550/572 nm to evaluate permeability.

### Enzyme-linked immunosorbent assay (ELISA)

TNF-α and IL-6 levels in rat brain and blood samples were quantified using ELISA kits (TNF-α: ERA56RBX5; IL-6: BMS625; Thermo Fisher, USA). Samples were appropriately diluted with coating buffer, and wells were blocked with 5% calf serum (F8318, MSK, Wuhan, China) at 37 °C for 40 min. Diluted samples were added to the wells, followed by incubation with enzyme-linked antibodies and substrate solution. The reaction was terminated by adding 50 μL stop solution to each well. Absorbance was recorded at 450 nm using a microplate reader (Bio-Rad, USA) within 20 min, and concentrations were calculated based on standard curve.

### Immunohistochemistry

To assess differentiation of NSCs into astrocytes, immunohistochemistry was performed using Nestin and GFAP antibodies. Paraffin-embedded brain sections (4 μm) were deparaffinized in xylene, rehydrated through graded ethanol, and subjected to antigen retrieval in citrate buffer (pH 6.0) by heating for 15 min. Endogenous peroxidase activity was blocked with hydrogen peroxide, followed by overnight incubation at 4 °C with primary antibodies against Nestin (14-5843-82, Thermo Fisher, USA) and GFAP (13-0300, Thermo Fisher, USA). After incubation with HRP-conjugated secondary antibodies, signals were developed using DAB substrate (Vector Laboratories, USA). Sections were counterstained with hematoxylin, and positive cells were quantified under a light microscope (Nikon, Japan).

### Statistical analysis

All data were analyzed using GraphPad Prism 9.5 (GraphPad Software, USA). Differences among multiple groups were assessed by one-way ANOVA followed by Tukey’s post hoc test, while comparisons between two groups were performed using two-tailed Student’s t-tests. Data are presented as mean ± standard deviation (SD), and experiments were repeated at least three times independently. A *p*-value < 0.05 was considered statistically significant (*p < 0.05, **p < 0.01, ***p < 0.001, ****p < 0.0001).

## Supplementary information

Supplementary Tables and figures.

The full uncropped Gels and Blots images.

## Data Availability

The RNA-seq data generated in this study have been deposited in the NCBI BioProject database under accession number PRJNA1463563 (https://www.ncbi.nlm.nih.gov/bioproject/PRJNA1463563). The full, uncropped original western blot images are included in the Supplementary Material. All other data generated or analyzed during this study are included in this article and/or its supplementary material files. Further enquiries can be directed to the corresponding author.
