# Supplementary material for: Transcriptomic analysis reveals that neural stem cell-derived exosomes regulate the HMGB1/TLR2 signaling axis to promote astrocytic differentiation and mitochondrial biogenesis in the repair of radiation-induced blood-brain barrier damage
Source: Cell Death Discov. 2026 Jul 25;12:373. doi: 10.1038/s41420-026-03250-4 (PMC13575103; doi:10.1038/s41420-026-03250-4)
Supplement: Supplementary file 1 [file 41420_2026_3250_MOESM1_ESM.docx]

**Table S1. Details of primary antibodies.**

| **Name** | **Cat. No** | **Dilution ratio** | **Manufacturer** |
| --- | --- | --- | --- |
| Alix | EPR23653-32 | 1:1000(WB) | Abcam |
| Hsp90 | #4874 | 1:1000(WB) | Cell signaling |
| CD63 | #10112 | 1:1000(WB) | Cell signaling |
| Tsg101 | #72312 | 1:1000(WB) | Cell signaling |
| BAX | #2772 | 1:1000(WB) | Cell signaling |
| BCL-2 | #15071 | 1:1000(WB) | Cell signaling |
| VEGF | JH121 | 1:500-1000 (WB) 1:20-1001:1000 (IF) | Invitrogen |
| MMP9 | #3852 | 1:1000(WB) | Cell signaling |
| Calnexin | ab22595 | 1:1000(WB) | Abcam |
| HMGB1 | ab18256 | 1:1000(WB) | Abcam |
| TLR2 | ab209216 | 1:1000(WB) | Abcam |
| NRF1 | ab175932 | 1:1000(WB) | Abcam |
| TFAM | MA5-16148 | 1/1000(WB) | Thermo Fisher |
| MFN2 | ab124773 | 1/1000(WB) | Thermo Fisher |
| PINK1 | ab186303 | 1:1000(WB) | Abcam |
| DNM1L | ab184247 | 1:1000(WB) | Abcam |
| GFAP | ab7260 | 1:1000(WB) | Abcam |
| STAT3 | ab68153 | 1:1000(WB) | Abcam |
| SOX9 | ab185966 | 1:1000(WB) | Abcam |
| Occludin | ab216327 | 1:1000(WB) | Abcam |
| Claudin-5 | ab131259 | 1:1000(WB) | Abcam |
| ZO-1 | ab307799 | 1:1000(WB) | Abcam |
| GAPDH | SAB4300645 | 1/1000(WB) | Sigma Aldrich |

**Table S2. RT-qPCR primer sequences.**

| **Gene** | **Primer** |
| --- | --- |
| *Tlr2* (Rat) | F 5’- GCCACAGGACTCAAGAGCAT -3’ |
|  | R 5’- AGAGATGCCTGGGCAGAATG -3’ |
| *Hmgb1* (Rat) | F 5’-TTAACAACACTTGGTGCTGGG-3’ |
|  | R 5’-CATTTTGGAAGCTGGTCCCC-3’ |
| *Gfap* (Rat) | F 5’-AGGCTAATGACTATCGCCGC-3’ |
|  | R 5’-GCCTCTCCAAGGACTCGTTC-3’ |
| *Stat3* (Rat) | F 5’- GGAAAGTATTGTCGCCCCGA-3’  R 5’- TGGTATTGCTGCAGGTCGTT-3’ |
| *Sox*9 (Rat) | F 5’- GCAAACACGTTGCAAATGGC-3’  R 5’- AACTCTGAAGGAGCCAAGCC-3’ |

Note: F, forward; R, reverse.

**Figure S1. Isolation and characterization of NSC-Exos.**

Note: (A) Schematic diagram of the exosome isolation process; (B) TEM image showing the double-membrane structure of exosomes (bar = 100 nm); (C) NTA of exosome size distribution; (D) Western blot analysis of exosome-specific markers (Alix, Hsp90, CD63, Tsg101) and the negative marker Calnexin. CL: cell lysate group; Exo: exosome group. Experiments were performed in triplicate.

**Figure S2. Analysis of uptake of derived EVs by cells and rat brain.**

Note: (A) Schematic of the experimental design: Exo-Green-labeled EVs were co-cultured with HNCs and NSCs, or intravenously injected into rats to assess the ability of EVs to cross the BBB and enter brain tissue; (B) Confocal microscopy images showing green fluorescence (Exo-Green) internalized in both HNCs and NSCs (indicated by white arrows), with nuclei stained with DAPI (blue) (bar = 10 μm); (C) MTT assay for cell viability; (D) IVIS imaging of rats at different time points demonstrating the progressive penetration and distribution of Exo-Green-labeled EVs in brain tissue up to 360 minutes after injection. All experiments were repeated three times. ns: no significant difference between groups.

**Figure S3. Analysis of key genes involved in mitochondrial function and interaction networks following exosome intervention.**

Note: (A) Heatmap illustrating differential expression of mitochondrial function-related genes between the IR and IR_Exo groups; (B) Correlation network depicting the relationships among DEGs, with red lines indicating positive correlations, blue lines indicating negative correlations, and line thickness representing the strength of correlation; (C) PPI network constructed using the STRING database, showing potential regulatory and interactive relationships between HMGB1, TLR2, and multiple mitochondrial function-related factors; (D) Schematic diagram summarizing the proposed mechanism: X-ray-induced brain injury activates the HMGB1/TLR2 signaling axis, which in turn suppresses protective responses such as mitochondrial fusion; (E) qPCR validation of the representative DEGs TNF and MT1 screened from the transcriptomic analysis in the IR and IR_Exo groups, showing that their expression trends are consistent with the RNA-seq results. IR: n = 3; IR_Exo: n = 3.

**Figure S4. TUNEL validation of neuronal apoptosis after HMGB1/TLR2 pathway intervention.**
Note: (A) Quantification of TUNEL-positive neuronal cells in the Control, IR, IR+Exo, IR+Exo+oeHMGB1, and IR+Exo+oeHMGB1+shTLR2 groups. (B) Representative TUNEL staining images showing neuronal apoptosis in each group. Data are presented as the mean ± SD from at least three independent experiments. Statistical significance was determined by one-way ANOVA followed by Tukey’s post hoc test. *****p* < 0.0001.

**Figure S5. Flow cytometric validation of astrocytic differentiation after HMGB1/TLR2 pathway intervention.**
Note: (A) Representative flow cytometry plots showing the proportion of GFAP-positive cells in the Control, IR, IR+Exo, IR+Exo+oeHMGB1, and IR+Exo+oeHMGB1+shTLR2 groups. (B) Quantification of GFAP-positive cells based on flow cytometry analysis. Data are presented as the mean ± SD from at least three independent experiments. Statistical significance was determined by one-way ANOVA followed by Tukey’s post hoc test. *****p* < 0.0001.
