# Supplementary material for: Transcriptomic analysis reveals that neural stem cell-derived exosomes regulate the HMGB1/TLR2 signaling axis to promote astrocytic differentiation and mitochondrial biogenesis in the repair of radiation-induced blood-brain barrier damage
Source: Cell Death Discov. 2026 Jul 25;12:373. doi: 10.1038/s41420-026-03250-4 (PMC13575103; doi:10.1038/s41420-026-03250-4)
Supplement: Supplementary file 2 [file 41420_2026_3250_MOESM2_ESM.docx]

Figure 1J-1

Figure 1J-2

Figure 1J-3

Figure 3C-1

Figure 3C-2

Figure 3C-3

Figure 4B-1

Figure 4B-2

Figure 4B-3

Figure 4B-4

Figure 4B-5

Figure 4B-6

Figure 5B-1

Figure 5B-2

Figure 5B-3

Figure 5B-4

Figure 6E-1

Figure 6E-2

Figure 6E-3

Figure 6E-4

Figure 7C-1

Figure 7C-2

Figure 7C-3

Figure 7G-1

Figure 7G-2

Figure 7G-3

Figure 7G-4

Figure 7G-5

Figure 7G-6

Figure 8C-1

Figure 8C-2

Figure 8C-3

Figure 8C-4

Figure S1D-1

Figure S1D-2

Figure S1D-3

Figure S1D-4

Figure S1D-5
